# ^1^H NMR-Based Metabolomics Reveals Refined-Huang-Lian-Jie-Du-Decoction (BBG) as a Potential Ischemic Stroke Treatment Drug With Efficacy and a Favorable Therapeutic Window

**DOI:** 10.3389/fphar.2019.00337

**Published:** 2019-04-12

**Authors:** Xiaowei Fu, Junsong Wang, Shanting Liao, Yan Lv, Dingqiao Xu, Minghua Yang, Lingyi Kong

**Affiliations:** ^1^Jiangsu Key Laboratory of Bioactive Natural Product Research and State Key Laboratory of Natural Medicines, School of Traditional Chinese Pharmacy, China Pharmaceutical University, Nanjing, China; ^2^Center for Molecular Metabolism, School of Environmental and Biological Engineering, Nanjing University of Science and Technology, Nanjing, China

**Keywords:** Refined-Huang-Lian-Jie-Du-Decoction, berberine, baicalin, geniposide, ischemic stroke, therapeutic window, metabolomics

## Abstract

Huang-Lian-Jie-Du-Decoction (HLJDD) is a traditional Chinese medicine (TCM) used to treat ischemic stroke. However, the complexity of its chemical composition makes quality control difficult. Berberine, baicalin, and geniposide are the three main ingredients in HLJDD. Here, a formula of BBG comprised of berberine, baicalin, and geniposide, known as Refined-Huang-Lian-Jie-Du-Decoction, was investigated for its efficacy, therapeutic window, and mechanisms of action. BBG was assessed on two major types of ischemic stroke, cerebral ischemia-reperfusion (I/R) injury, and continuous ischemia injury, respectively. BBG showed efficacy comparable to HLJDD in the treatment of cerebral I/R injury within 5 h after injury initiation but did poorly in treating continuous ischemia injury. BBG exhibited neuroprotective effects on cerebral I/R injury by regaining the balance in energy metabolism, oxidative stress, amino acid metabolism, inflammation, and nucleic acid metabolism. These results suggested that BBG could be a good alternative to HLJDD, with high efficacy and a long therapeutic window, which shows great potential for drug development to treat stroke.

## Introduction

Stroke remains a global health problem, particularly in undeveloped countries (O'donnell et al., 2016). It is the second cause of death clinically (Lozano et al., 2012), making its prevention and treatment a worldwide health concern. Ischemic stroke, accounting for over 80% of stroke cases, is formed by a sudden obstruction of blood vessels in the brain, which soon results in neuronal death if left without intervention (Donnan et al., 2008). Clinically, ischemic stroke is divided into two major types: cerebral ischemia-reperfusion (I/R) and continuous ischemia, which could be induced by transient middle cerebral artery occlusion (TMCAO) and permanent middle cerebral artery occlusion (PMCAO) surgeries in rats (Matucz et al., 2006). Up to now, only tissue plasminogen activator (t-PA) has been approved by the Food and Drug Administration (FDA, USA). However, it only benefits about 5% of patients due to its high risk-to-benefit ratio and narrow therapeutic window (Mozaffarian et al., 2016). Therefore, a high priority in the field is to seek other therapies with good efficacy and safety.

Huang-Lian-Jie-Du-Decoction (HLJDD), a well-known traditional Chinese medicine (TCM) consisting of Rhizoma Coptidis, Radix Scutellariae, Cortex Phellodendri, and Fructus Gardenia, has widely been applied to treat cerebrovascular diseases and ischemic stroke in many Asian countries for centuries (Kondo et al., 2000; Xu et al., 2000). However, HLJDD is extremely complex in its components, making its quality control difficult. Many studies have demonstrated that berberine, baicalin, and geniposide, the three major ingredients in HLJDD, can each exert preventive effects against ischemic stroke individually (Ahmed et al., 2015; Huang et al., 2017; Liang et al., 2017; Zhang et al., 2017a). In this study, we aim to answer the questions on whether component combination of berberine, baicalin, and geniposide (BBG), namely Refined-Huang-Lian-Jie-Du-Decoction, could achieve an efficacy less than, equal to, or better than the formula and whether unexpected effects, e.g., toxicity, occur due to this combination. This is important for the development of new drugs based on traditional therapies.

Metabolomics is a thriving technology defined as the quantitative analysis of dynamic metabolic responses to pathophysiological stimuli or genetic modification (Nordström and Lewensohn, 2010; Baker, 2011). Metabolomics provides a new perspective on the understanding of multifactorial mechanisms of diseases. It is a feasible approach for the assessment of the drug treatment or intervention effects.

The therapeutic window is especially important for the treatment of ischemic stroke (Xu et al., 2006; Zeynalov et al., 2017). The committee of the Stroke Therapy Academic Industry Roundtable (STAIR) proposed that the timing of drug administration is highly correlated with the neuroprotective effects of a treatment, and future therapies should emphasize clarification of the therapeutic window for a better clinical guide (Fisher et al., 2007, 2009). However, many neuroprotective drugs, such as N-methyl-D-aspartate (NMDA) antagonists or lercanidipine (a calcium channel blocker), exhibit protective effects when the treatment takes place within a short time immediately after stroke occurrence (Gupta et al., 2017). Given their short therapeutic window, these drugs have limited clinical applicability. Therefore, we were interested in finding a satisfactory therapeutic window and efficacy for BBG on two types of ischemic stroke, including cerebral I/R injury (by TMCAO model) and continuous ischemia injury (by PMCAO model). The therapeutic window and mechanisms of BBG were examined by a ^1^H NMR-based metabolomics approach, complemented with histology, immunohistochemistry, and biochemical evaluation.

## Materials and Methods

### Chemicals and Reagents

Berberine (>98.0% purity) and geniposide (>98.0% purity) was obtained from Qingdao Jieshikang Biotech Co., Ltd. (Qingdao, China), and baicalin (>98.0% purity) was obtained from Dalian Meilun Biotech Co., Ltd. (Dalian, China). Four kinds of herbs in HLJDD, including Rhizoma Coptidis (*Coptis chinensis* Franch, Ranunculacea), Radix Scutellariae (*Scutellaria baicalensis* Georgi, Labiatae), Cortex Phellodendri (Phellodendron chinensis Schneid, Rutaceae), and Fructus Gardeniae (*Gardenia jasminoides* Ellis, Rubiaceae), were provided by the Jiangsu Medicine Company (Nanjing, China, Drug GMP certificate: SUJ0623. Drug Manufacturing Certificate: SUY20110051) and authenticated by Professor Mian Zhang, Department of Medicinal Plants, China Pharmaceutical University, Nanjing, China. All herbs were processed in conformity to standards of Chinese Pharmacopeia 2015. Sodium 3-(trimethylsilyl)-propionic acid (TSP), pyridine (99.8% GC), 2, 3, 5-triphenyltetrazolium chloride (TTC) and deuterium oxide (D_2_O, 99.9%) were bought from Sigma-Aldrich (St. Louis, MO, USA). The enzyme-linked immunosorbent assay (ELISA) kits for TNF-α, IL-1β, and IL-6 in rats, were provided by Senbeijia Bioengineering Institute (Nanjing, China). The test kits for superoxide dismutase (SOD), malondialdehyde (MDA), nitric oxide (NO), catalase (CAT), glutathione (GSH), glutathione disulfide (GSSG), glutathione peroxidase (GSH-PX), and glutathione reductase (GR) were purchased from Nanjing Jiancheng Bioengineering Institute (Nanjing, China).

**Figure 1:**
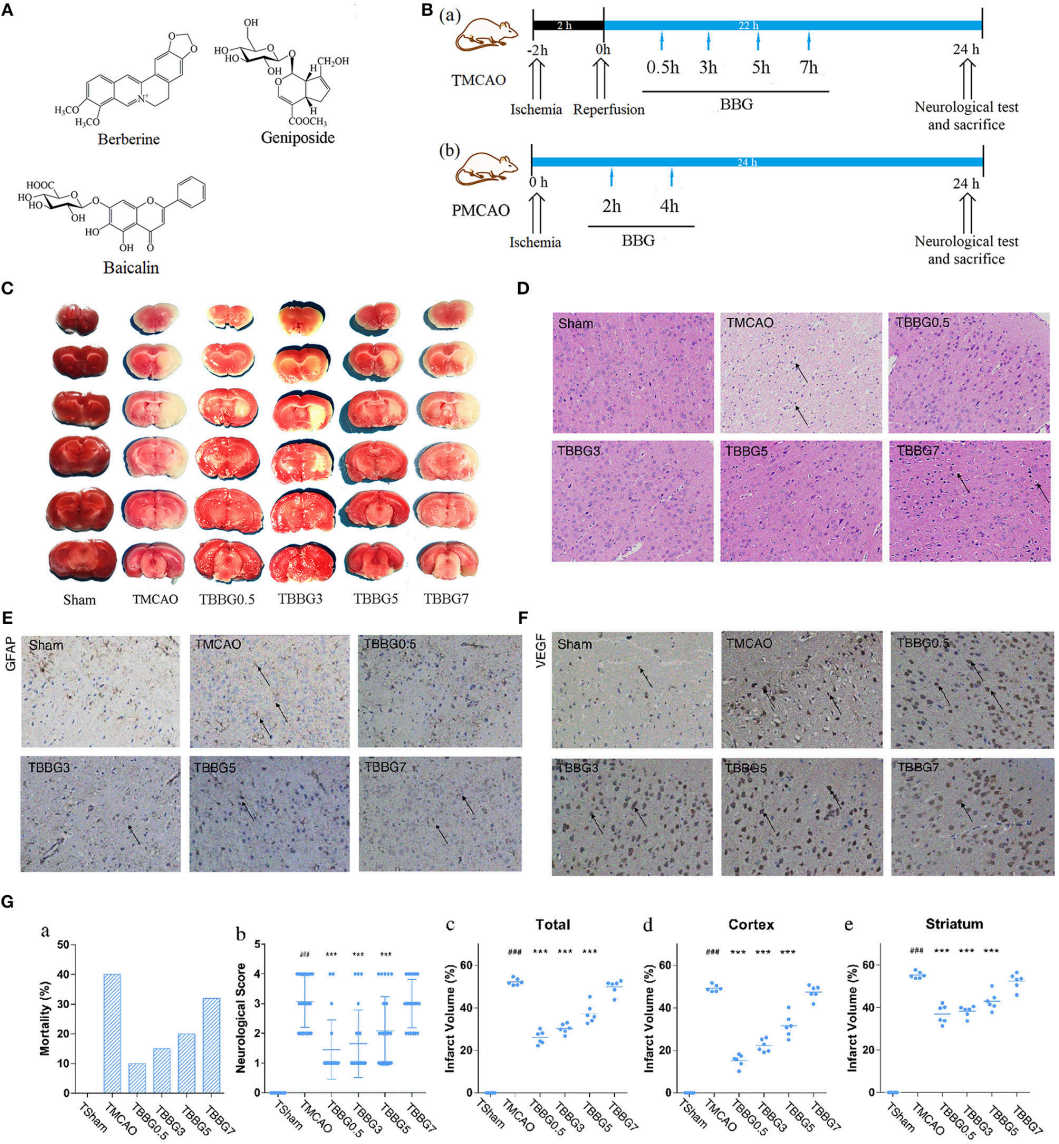
Experimental design and neuroprotective effects of BBG in TMCAO rats. Chemical structures of berberine, baicalin, and geniposide **(A)**. Schematic protocol of surgery for ischemic stroke with BBG **(B)** for TMCAO, the rats were subjected to 2-h ischemia and 22-h reperfusion and treated by BBG at 0.5, 3, 5, or 7 h (TBBG0.5, TBBG3, TBBG5, and TBBG7 group, respectively) after reperfusion **(B**a**)**; for PMCAO, the rats were subjected to 24-h ischemia and treated by BBG at 2 or 4 h (PBBG2 and PBBG4 group, respectively) after onset **(B**b**)**. TTC staining of brains (*n* = 6) **(C)**. HE staining of brain tissues to reflect neuronal loss (×200, *n* = 4) **(D)**. Immunohistochemical staining of brain tissues to show the expressions of GFAP and VEGF (×200, *n* = 4) **(E,F)**. Morality, neurological score and infarct volume of each group **(G)**. ^#^*p* < 0.05, ^##^*p* < 0.01, and ^###^*p* < 0.001 MCAO group vs. Sham group; *p < 0.05, **p < 0.01, and ***p < 0.001 treatment groups vs. TMCAO group.

BBG was prepared by combining berberine, baicalin, and geniposide in a ratio of 5.05:4.02:2.70, to match their proportions in the HLJDD formula: 5.05, 4.02, and 2.70%, respectively (Figure 1A; Zhu et al., 2013). That is to say, each gram of BBG contains 430 mg of berberine, 340 mg of baicalin, and 230 mg of geniposide. HLJDD was prepared by mixing its four component herbs, Rhizoma Coptidis, Radix Scutellariae, Cortex Phellodendri, and Fructus Gardeniae at the weight of 300, 200, 200, 300 g (3:2:2:3). The mixture was extracted with water (1:10, w/v) three times at 1 h each. The solution was mixed and lyophilized to afford an extract of HLJDD (250.1 g, yield: 25.01%). Prior to use, BBG and HLJDD were suspended in 0.5% CMC-Na (carboxymethylcellulose sodium salt) to a final concentration of 50 and 425 mg/ml, respectively.

### Animals

Healthy male Sprague-Dawley (SD) rats (250 ± 10 g) were obtained from Sipper-BK Laboratory Animals Co., Ltd. (Shanghai, China). The house condition for rats was maintained at 25°C with 40–60% of humidity and a 12/12 h light/dark cycle. The rats were allowed to acclimatize for 7 days before experiments. All procedures were in compliance with the Animal Ethics Committee of China Pharmaceutical University. Before experiments, rats were deprived of food for 12 h but had continued access to water.

### The TMCAO and PMCAO Models

Cerebral ischemia-reperfusion injury was induced by TMCAO surgery, as previously described, with slight modifications (Longa et al., 1989; Wang et al., 2014). After administering anesthesia with chloral hydrate (3.5%, 350 mg/kg, i.p.), a silicon-coated 4-0 suture (Guangzhou Jialing Biotechnology Co., Ltd., Guangzhou, China) was inserted to occlude the origin of the middle cerebral artery (MCA) through the external carotid artery (ECA) and internal carotid artery (ICA). The rectal temperature of the rats was kept at 37°C and the occlusion and reperfusion were assessed by a laser Doppler flowmeter. Finally, the right MCA was occluded for 2 h with subsequent 22-h reperfusion by the withdrawal of the inserted filament.

Continuous ischemia injury was induced by PMCAO surgery (Yang et al., 2012). The protocol used was the same as described above, except that the MCA was treated with a suture for 24 h.

### Drug Treatments

The dosage of BBG was optimized to 500 mg/kg (containing 215 mg/kg of berberine, 170 mg/kg of berberine, and 115 mg/kg of geniposide) in a preliminary experiment (Figure S1). For TMCAO, BBG was intragastrically (i.g.) administered at 0.5, 3, 5, or 7 h (TBBG0.5, TBBG3, TBBG5, or TBBG7 groups, respectively) after the reperfusion, followed by a second gavage 4 h later (Figure 1B). For PMCAO, BBG was administered at 2 or 4 h (PBBG2 and PBBG4 groups) after occlusion of the MCA, followed by a second gavage 4 h later. CMC-Na solution (0.5%, 10 ml/kg) was administered intragastrically (i.g.) at 2 h after the surgery. To compare the efficacy of BBG, HLJDD was intragastrically (i.g.) administered to rats at 4.25 g/kg (this dosage contained 215 mg/kg of berberine, 170 mg/kg of baicalin and 115 mg/kg of geniposide according to their ratios). Rats were divided into different groups randomly.

### Evaluation of Mortality, Neurological Defects, and Infarct Volume

The mortality rate was calculated at 24 h after MCAO, as an essential parameter to study the therapeutic window. Moreover, the neurological function of each rat was tested as previously described (Wang et al., 2014). The neurological scores were determined according to Longa's five-point scale: 0, rats behave normally; 1, rats cannot stretch left forelimb; 2, rats circle to the opposite side; 3, rats fall to the opposite side; and 4, rats lose consciousness. After neurological function testing, rats were sacrificed. Brain infarct volumes were determined using the TTC (Sigma) staining method (Bederson et al., 1986). After being sacrificed, rat brains were stored at −20°C for 30 min and sliced into six 2-mm-thick slices. The slices were incubated with 1% TTC at 37°C for 15 min and fixed with 4% formaldehyde overnight. Non-infarcted regions were stained red while infarcted regions were stained white. The percentage of infarcted volumes (*I*%) was calculated by Image J (NIH, USA) blindly, using the equation below:

$$\begin{array}{l} I\%=\left(Vc-Vi\right)/Vc\times \;100\% \end{array}$$

*V*c = volume of non-infarcted regions in the left hemisphere

*V*i = volume of non-infarcted regions in the right hemisphere (Zhang et al., 2006)

### Histopathology Examination and Immunohistochemistry

Fresh brain tissues were soaked in 4% paraformaldehyde for 24 h and then embedded in paraffin. The brain sections were stained with hematoxylin and eosin (H&E) and inspected using light microscopy (×200, Olympus DX45) by a pathologist. For immunohistochemical examination, formalin-fixed, and paraffin embedded brain tissue sections were employed and then activities of glial fibrillary acidic protein (GFAP) and vascular endothelial growth factor (VEGF) were detected by Goodbio Technology Co., Ltd. (Nanjing, China). The staining was photographed under light microscopy and analyzed by Image-Pro Plus (Version 6.0).

### Biochemistry and ELISAs

Brain tissue from the right hemisphere was collected and homogenized in 0.86% normal saline. The supernatant of brain extracts and serum samples were collected and stored in liquid nitrogen. The levels of oxidative stress-related indexes in brain tissues, including SOD, MDA, NO, CAT, GSH-PX, GSH, GSSG, and GR were quantified using commercially available kits. The amount of TNF-α, IL-1β, and IL-6 in serum samples was determined using ELISA kits according to the manufacturer's instructions.

### Metabolomics Research by ^1^H NMR

#### Sample Preparation

The frozen ischemic hemisphere (500 mg) was homogenized by a TissueLyser (JXFSTPRP-24, Shanghai Jingxin Industrial Development Co., LTD) in 50% acetonitrile (5 ml g^−1^ tissue). The homogenate was centrifuged for 10 min at 4°C and 12,000 rpm. The supernatant was then lyophilized under nitrogen. Dried brain extract was dissolved in 600 μl D_2_O phosphate buffer (0.2 M, pH 7.4, containing 0.05% TSP) and transferred into a 5 mm NMR tube.

For the serum samples, methanol (700 μl) was added to the serum (300 μl) and the mixture was vortexed for 3 min to precipitate the protein. Next, samples were centrifuged for 10 min at 4°C and 12,000 rpm. The supernatant was lyophilized under nitrogen. Dried serum extracts were dissolved in 550 μl D_2_O phosphate buffer and transferred into NMR tubes for NMR analysis.

#### ^1^H NMR Spectroscopy Analysis

^1^H NMR spectra of brain and serum samples were obtained at 298 K on a 600 MHz NMR spectrometer (Bruker Avance III, Germany) coupled to a 5 mm TCI cryoprobe. A nuclear overhauser effect spectroscopy pulse sequence (relaxation delay-90°-μs-90°-tm-90°-acquire-FID) was employed to moderate the H_2_O signal. ^1^H NMR spectra were obtained with 128 scans into 32 K data points within the range of 10,000 Hz. The FIDs were weighted using an exponential window function with a line-broadening of 0.3 Hz before a Fourier transformation.

#### Spectral Pre-processing

All spectra were referenced to TSP (0 ppm). Corrections of phase and baseline were performed with the Bruker Topspin software (version 3.0). Spectra were automatically converted to ASCII files using MestReNova (version 8.0.1, Mestrelab Research SL), and then imported into R software for alignment, using an in-house developed R-script, to further alleviate phase and baseline distortions. Spectra from 0 to 10 ppm, except within the region of residual H2O (4.65–5.25 ppm), were segmented into 0.0025 ppm integrated spectral buckets (De Meyer et al., 2008). To address solvent dilution effects, the spectral buckets were modified by probabilistic quotient normalization and Pareto scaling, prior to principal component analysis (PCA).

#### Identification of Metabolites

**Figure 2:**
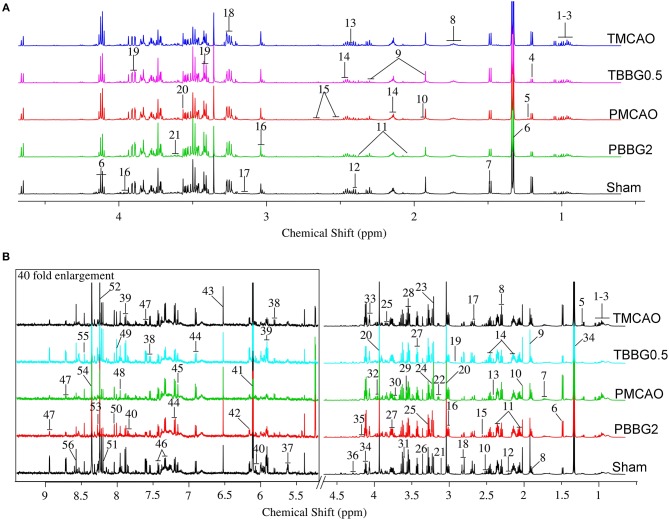
The typical 600 MHZ 1H NMR spectra for serum **(A)** and brain **(B)** with the metabolites labeled. Serum: 1. Leucine (Leu); 2. Valine (Val); 3. Isoleucine (Ile); 4. 3-Hydroxybutyrate (3-HB); 5. Methylmalonate (MMA); 6. Lactate (Lac); 7. Alanine (Ala); 8. Lysine (Lys); 9. Gamma-aminobutyric acid (GABA); 10. Acetate (AC); 11. Glutamate (Glu); 12. Pyruvate (Pyr); 13. Succinate (Suc); 14. Glutamine (Gln); 15. Citrate (Cit); 16. Creatine (Cr); 17. Ethanolamine (Eta); 18. Choline (Cho); 19. Glucose (Glc); 20. Glycine (Gly); 21. Glycerol (Gyo). Brain: 1. Leucine (Leu); 2. Valine (Val); 3. Isoleucine (Ile); 4. 3-Hydroxybutyrate (3-HB); 5. Methylmalonate (MMA); 6. Alanine (Ala); 7. Lysine (Lys); 8. Gamma-aminobutyric acid (GABA); 9. Acetate (AC); 10. N-Acetylaspartate (NAA); 11. Glutamate (Glu); 12. N-Acetylglutamate (NAG); 13. Succinate (Suc); 14. Glutamine (Gln); 15. Citrate (Cit); 16. Glutathione (GSH); 17. Methionine (Met); 18. Aspartate (Asp); 19. Trimethylamine (TMA); 20. Creatine/Creatine phosphate (Cr/PCr); 21. Malonate (Mal); 22. Ethanolamine (Eth); 23. Choline (Cho); 24. Trimethylamine-N-oxide (TMAO); 25. Betaine (Bet); 26. Taurine (Tau); 27. Glucose (Glu); 28. Myo-inositol (Myo); 29. Glycine (Gly); 30. Glycerol (Gyo); 31. Threonine (Thr); 32. Serine (Ser); 33. Ascorbate (Asc); 34. Lactate (Lac); 35. O-phosphocholine (OPC); 36. Inosine (Ino); 37. UDP-glucose (UDP-glc); 38. Uracil (Ura); 39. Uridine (Uri); 40. Cytidine (Cyt); 41. ADP; 42. AMP; 43. Fumarate (Fum); 44. Tyrosine (Tyr); 45. Anserine (Ans); 46. Phenylalanine (Phe); 47. Nicotinurate (Nic); 48. Xanthine (Xan); 49. Guanosine (Gua); 50. Carnosine (Carn); 51. Hypoxanthine (Hyp); 52. Oxypurinol (Oxy); 53. Adenosine (Ade); 54. NADH; 55. Formate (For); 56. ATP.

Representative 600 MHz ^1^H NMR spectra from the serum and brain of the TMCAO, PMCAO, TBBG0.5, PBBG2, and Sham groups are shown in Figure 2. Metabolites were identified by querying public metabolomics databases, such as MMCD (http://mmcd.nmrfam.wisc.edu/) and HMDB (http://www.hmdb.ca/). The Chenomx NMR suite software (version 8.1, Canada) was also used to confirm metabolites. Identified metabolites in the serum and brain are displayed in Tables S1, S2, respectively.

### Multivariate and Univariate Data Analysis

Standard NMR data were processed by PCA and orthogonal partial least squares-discriminant analysis (OPLS-DA) using the R software. PCA, which is unsupervised and involves no group information of each subject, was used to describe metabolic patterns and detect outliers among brain or serum samples. The PCA score plots showed obvious differences between the Sham and TMCAO/PMCAO groups (Figure S2). Lacking in group information, the PCA is less able to capture and classify variables than supervised OPLS-DA. OPLS-DA discriminates the first component contributing to the grouping from the subsequent components containing irrelevant variations. The results are presented with score plots, to visualize the classification, and with color-coded loading plots, to reveal obviously varied metabolites. The red-colored variables in the loading plots contributed more to grouping than the blue-colored variables. The S-plot helped to visualize metabolites that were distinctive both statistically and biochemically, according to their reliability and contribution to the classification. All OPLS-DA models were verified by *R*^2^ and *Q*^2^ parameters to assess goodness of fit and prediction quality. The fold-change (FC) value for each metabolite and the corresponding *p*-value calculated by the Benjamini-Hochberg procedure, were all presented in colored tables.

### Shared and Unique Structure Analysis, and Venn Analysis

The shared and unique structure (SUS) plot is an extension of S plot applied to further compare the effects two treatment groups (e.g., TBBG0.5 vs. TBBG3, TBBG0.5 vs. TBBG5, and TBBG0.5 vs. TBBG7) using the same reference group (TMCAO group). The SUS plot showed the correlation between groups, so it should be located within −1 and +1 for both the X and Y axes. A compound was considered important to drug action if the variable influence on projection (VIP) was beyond 1. Metabolites close to the colored diagonal stood for the shared compounds. The red and green color of the diagonal represented a positive and negative correlation, respectively. Metabolites scattered in the colored frames were unique for a specific group. Finally, the different regions in the SUS plots were visualized in Venn plots for further investigation.

### Correlation Network Analysis

The correlation networks among metabolites from all groups were established based on Pearson correlation coefficients. The coefficients beyond a value of 0.6 between two metabolites were presented with dotted lines to reflect correlations, while the coded color and width of the correlated lines indicated calculated values of metabolic correlations. The networks were additionally mapped to the KEGG database to supply biochemical relationships (indicated by gray arrows) between unmeasured and detected metabolites.

### Statistical Analysis

The results were presented as mean ± standard deviation (S.D.). A Student's *t*-test was adopted for a comparison between two groups. One-way ANOVA followed by Tukey's multiple comparison tests was used to determine differences when the data involved three or more groups. *P* < 0.05 was considered statistically significant.

## Results

### BBG Mimicked HLJDD in the Delayed Treatment of Ischemic Stroke

TTC staining results revealed that the infarct volumes in the BBG treated groups were significantly reduced when compared to those of the TMCAO group, especially in 500 and 1,000 mg/kg groups (Figure S1). The dosage of 500 mg/kg is optimal in consideration of the efficacy and dose. There was no significant difference in the infarct volumes between the HLJDD and BBG groups (Figure S3). Besides, treatments with each BBG component (berberine, baicalin, or geniposide) individually could obviously reduce infarct volumes when compared to TMCAO group, but not as prominently as with BBG (*P* < 0.05).

### BBG Reduced Mortality, Neurological Defects, and Cerebral Infarction of TMCAO Rats

Mortality and neurological scores in the TMCAO group increased significantly when compared to the Sham group, indicating that cerebral I/R injury was successfully induced (Figures 1Ga,b). In the TBBG0.5/3/5 groups, mortality and neurological scores were significantly reduced compared to those of the TMCAO group, while the TBBG7 group showed no difference in mortality or neurological scores.

According to the results of TTC staining, severe infarct volumes (white region in brain slices) were observed in the TMCAO group and were calculated finally (Figures 1C,Gc–e). Infarct volumes were significantly decreased when the BBG treatment was at 0.5, 3, and 5 h after reperfusion. However, no significant decrease in the infarcted region was observed when the treatment was delayed to 7 h. It is worth noting that that remarkable salvage in cortex were observed according to the sub-analysis.

### BBG Ameliorated Histopathological Injury of Ischemic Brain in TMCAO Rats

Compared with the Sham group, obvious pathological lesions were observed in the ischemic brains of TMCAO rats. The integrity of the neuronal structure was destructed along with the loss of neurons and the appearance of many vacuolated spaces. In I/R injury, treatment with BBG within 5 h after reperfusion significantly reduced pathological lesions in ischemic brains of TMCAO rats, but these pathological abnormalities persisted when the administration was delayed by 7 h (Figure 1D).

The expression levels of GFAP and VEGF were significantly increased in TMCAO rats. In cerebral I/R injury, BBG treatment at 0.5, 3, and 5 h after TMCAO notably reduced the expression levels of GFAP and VEGF, while it made no difference when delayed by 7 h (Figures 1E,F).

### BBG Exhibited Outstanding Anti-oxidative and Anti-inflammatory Effects for TMCAO Injury

**Figure 3:**
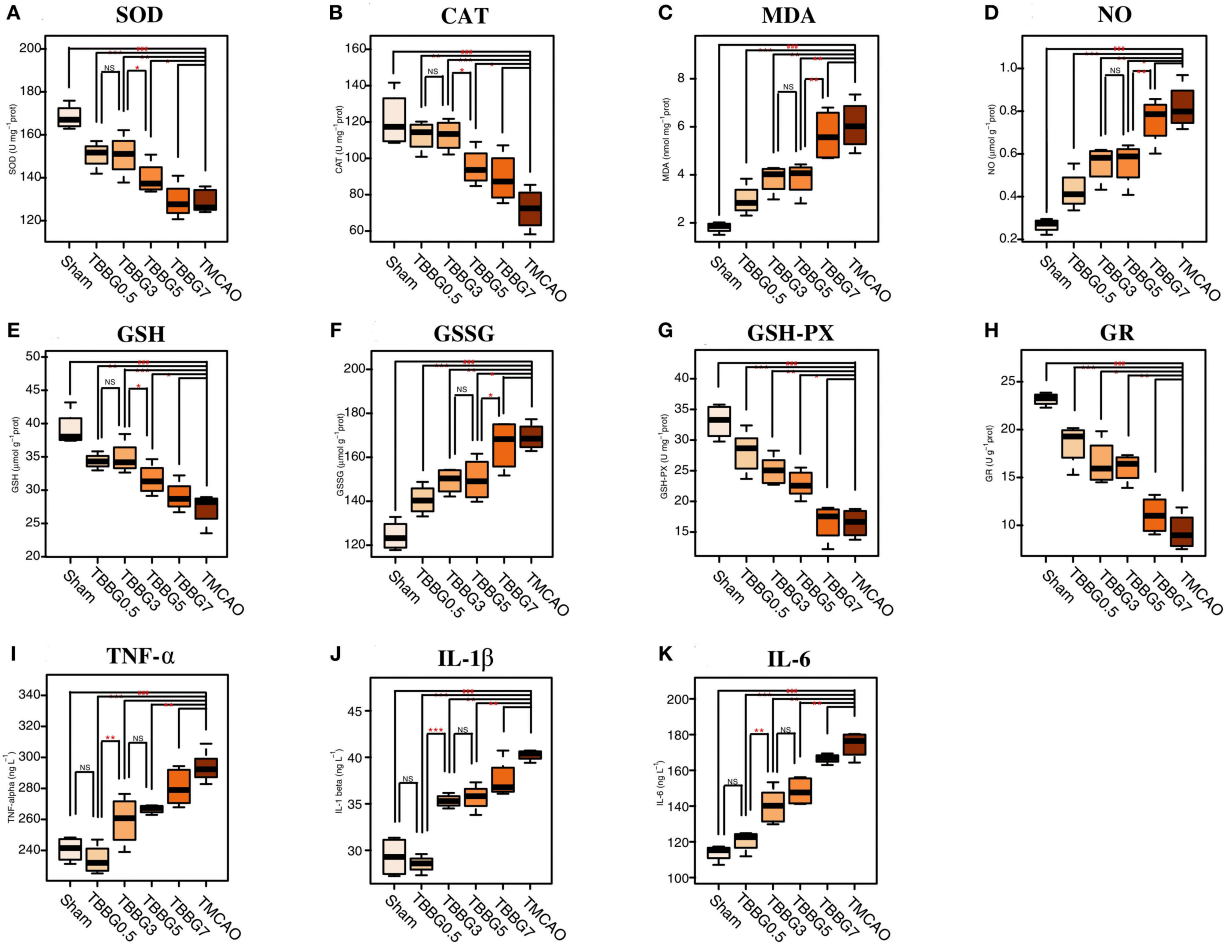
Boxplots for oxidative stress indexes and inflammatory cytokines. The levels of biochemical indexes SOD, CAT, MDA, NO, GSH, GSSG, GSH-Px, and GR **(A-H)** in ischemic brains of the Sham, BBG treatment and TMCAO groups (*n* = 4). The levels of inflammatory cytokines TNF-α, IL-1β, and IL-6 **(I-K)** in serum of the Sham, BBG treatment and TMCAO groups (*n* = 4). All data are expressed as mean ± S.D. ^###^*p* < 0.001 for TMCAO group vs. Sham group; *p < 0.05, **p < 0.01 and ***p < 0.001 for treatment groups vs. TMCAO group or treatment groups; NS: no significant differences between groups.

The oxidative stress-related biochemical indexes and levels of inflammatory cytokines were determined (Figure 3). The levels of MDA and NO significantly increased in the TMCAO group and were decreased in the TBBG0.5, TBBG3, and TBBG5 groups; but not in the TBBG7 group. The TMCAO group also exhibited a notable reduction in GSH levels and a significant accumulation of GSSG, along with an inhibition of GSH-PX and GR; again, these changes were markedly relieved by BBG, but only in the TBBG0.5, TBBG3, and TBBG5 groups. Activity levels of the antioxidants SOD and CAT were remarkably inhibited in the TMCAO group, an effect that was significantly reversed in the TBBG0.5, TBBG3, and TBBG5 groups. Meanwhile, TMCAO caused significant increases in inflammatory cytokines, such as TNF-α, IL-1β, and IL-6. The levels of these inflammatory cytokines decreased markedly in the TBBG0.5, TBBG3, and TBBG5 groups. No significant improvement was observed in the TBBG7 when it was compared with the TMCAO group.

### BBG Reversed Disturbances in the Metabolic Patterns of TMCAO Rats

#### Serum Metabolomic Analysis

**Figure 4:**
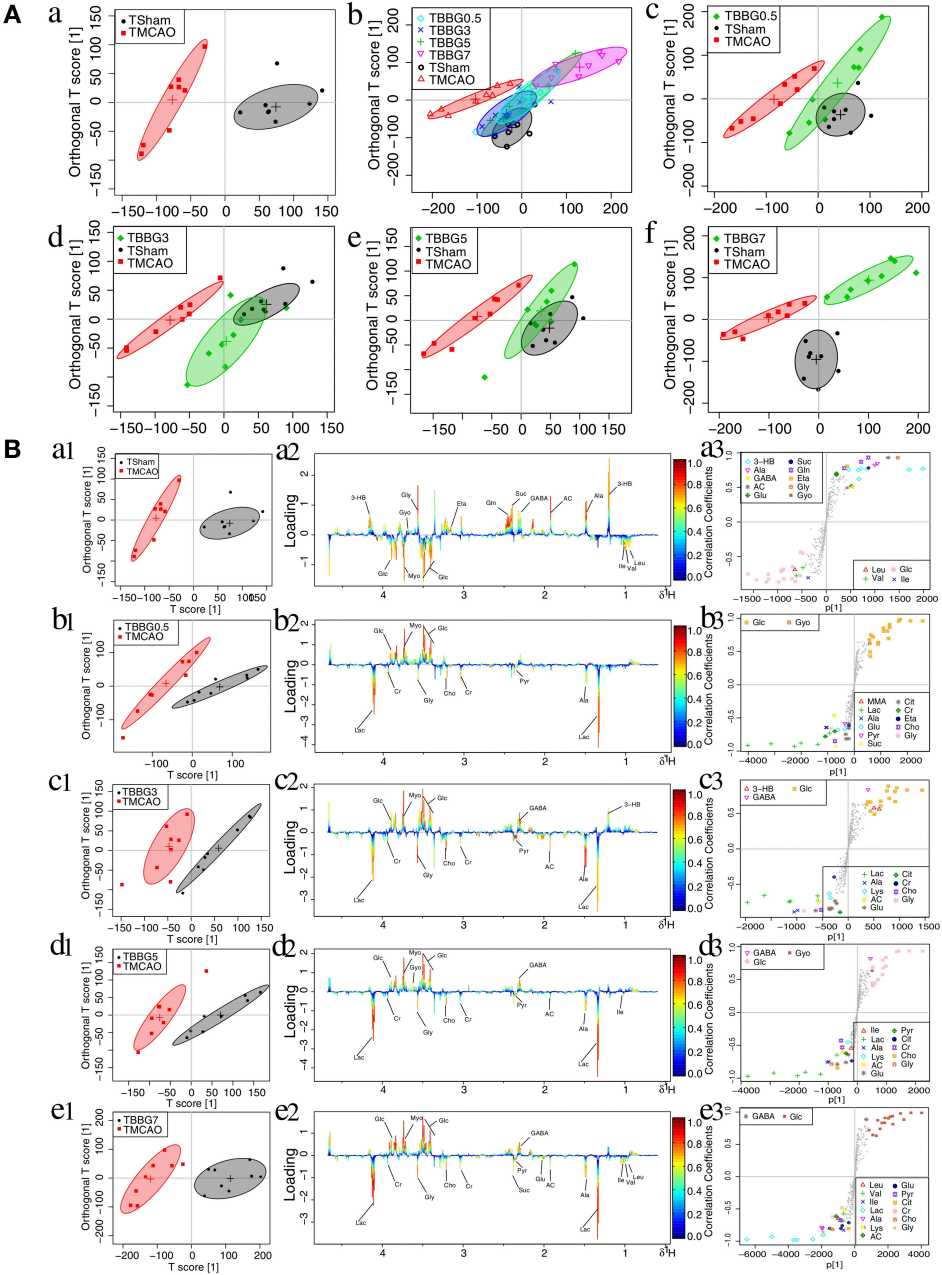
OPLS-DA analysis of serum from TMCAO treatment groups based on ^1^H NMR data. Score plots of OPLS-DA analysis based on 1H NMR from serum samples of Sham (TSham), TMCAO, TBBG0.5, TBBG3, TBBG5, and TBBG7 groups, where one point represents one sample and one ellipse corresponds to a confidence interval of 95% stood for a grouping (*n* = 8) **(A)**. Color-coded loading plots and S-plot for OPLS-DA analysis in serum samples **(B)**. In the loading plots, the color bar represents the weight of variables contributing to grouping: significant in red and insignificant in blue. In the S-plot, the points represent different variables.

In the OPLS-DA score plots of serum samples, the metabolic patterns of the TBBG0.5, TBBG3, and TBBG5 groups were clearly distinguishable from that of the TMCAO group and partially overlapped with that of the Sham group (Figure 4A). Nevertheless, the global metabolic patterns of the TBBG7 group were clearly distinct from those of the Sham group.

The ^1^H NMR metabolomics data of the TMCAO group were compared with those of the Sham/TBBG0.5/TBBG3/TBBG5/TBBG7 groups individually to identify significant metabolites (Figure 4B). In the score plot, the Sham and TMCAO groups showed clear separation with a perfect goodness of fit (*R*^2^*Y* = 0.92, *Q*^2^*Y* = 0.82, Figure S4A), suggesting the establishment of an excellent TMCAO model. In the TMCAO group, as compared with the Sham group, color-coded loading plots and S-plots showed evident increases in leucine, valine, isoleucine, and glucose, and significant decreases of 3-hydroxybutyrate, alanine, GABA, acetate, succinate, glutamine, and glycine. A clear separation of serum metabolic patterns was observed between the TMCAO group and the TBBG0.5/TBBG3/TBBG5 groups with high *Q*^2^ values. OPLS-DA loading plots and S-plots coupled with a color-coded table (Table 1) revealed that metabolic disturbances in serum could be ameliorated in the TBBG0.5, TBBG3, and TBBG5 groups, but not the TBBG7 group.

**Table 1:**
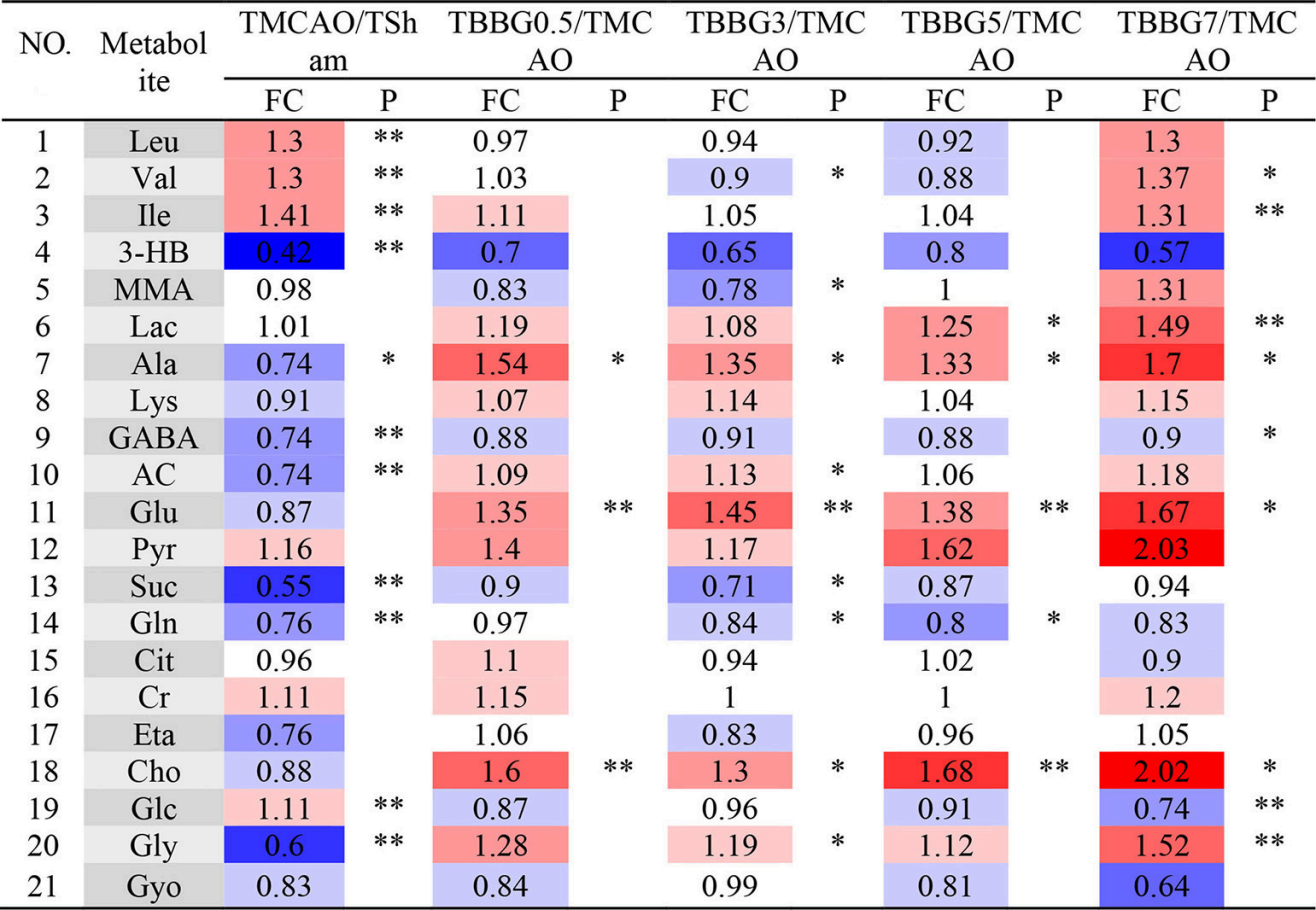
Potential marker metabolites and their fold changes^a^ among TMCAO treatment groups and the associated *P*-values^b^ in serum based on ^1^H NMR.

a *Color coded according to the fold change (FC), red represents higher and blue represents lower concentrations of metabolites. Color bar 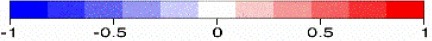
*.

b *P-values corrected by Benjamini–Hochberg methods were calculated based on a parametric Student's t-test or a nonparametric Mann–Whitney test*.

^*^p < 0.05,

^**^p < 0.01.

#### Brain Metabolomic Analysis

**Figure 5:**
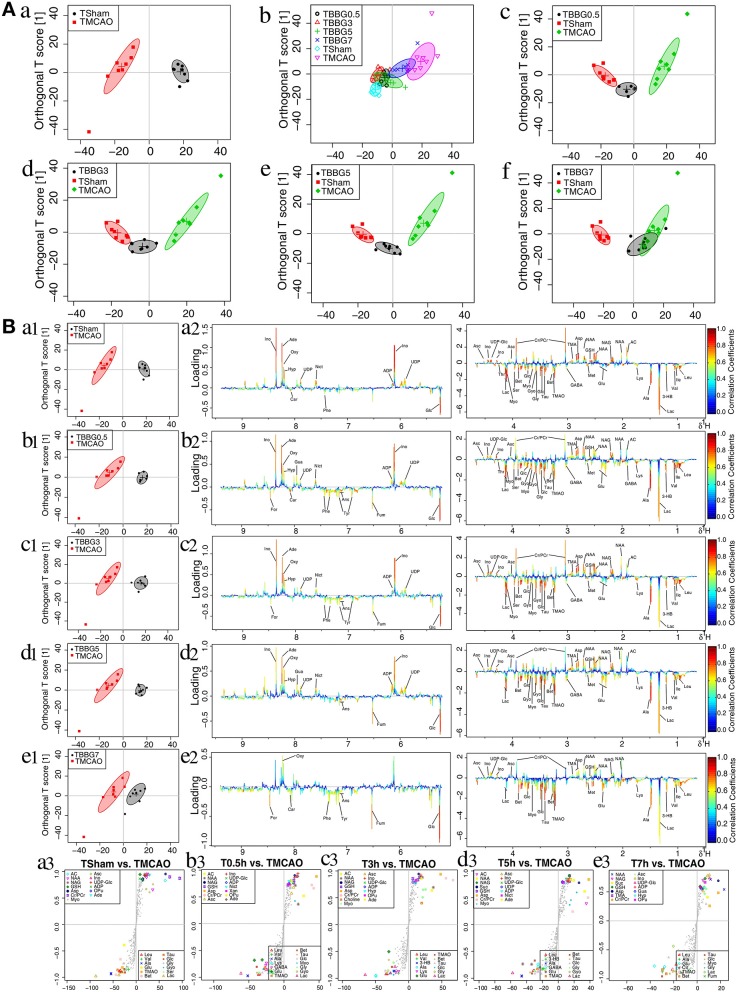
OPLS-DA analysis of brains from TMCAO treatment groups based on ^1^H NMR data. Score plots of OPLS-DA analysis based on ^1^H NMR from brain samples of Sham (TSham), TMCAO, TBBG0.5, TBBG3, TBBG5, and TBBG7 groups (*n* = 8) **(A)**. Color-coded loading plots and S-plot for OPLS-DA analysis in serum samples **(B)**.

The data from brain samples of the Sham, TMCAO, and different treatment groups were analyzed by OPLS-DA to investigate the BBG efficacy in different therapeutic windows on TMCAO rats. According to the OPLS-DA results (Figure 5A), the Sham and TMCAO groups were far from each other with the TBBG0.5, TBBG3, and TBBG5 groups closing to the Sham group in the score plots. However, the TBBG7 group was overlapped with the TMCAO and far from the Sham group

To investigate metabolic disorders in TMCAO brains and to reveal therapeutic effects of related metabolites, TMCAO rats were compared with the Sham, TBBG0.5, TBBG3, TBBG5, and TBBG7 groups respectively (Figure 5B). The brain score plots exhibited an obvious separation between the Sham and TMCAO groups with an ideal goodness of fit (*R*^2^*Y* = 0.95, *Q*^2^*Y* = 0.81, Figure S4C), indicating an excellent model. The loading plots and S-plots revealed that the TMCAO group had increased levels of leucine, valine, isoleucine, 3-hydroxybutyrate, alanine, lysine, glutamate, citrate, methionine, trimethylamine N-oxide, glucose, glycine, glycerol, threonine, serine, lactate, O-phosphocholine, and guanosine; in contrast, it had significantly decreased levels of acetate, N-acetylaspartate, N-acetylglutamate, glutathione, aspartate, trimethylamine, creatine/creatine phosphate, malonate, inosine, UDP-glucose, UDP, ADP, nicotinurate, xanthine, hypoxanthine, oxypurinol, and adenosine. Clear separations were also observed between the TMCAO and TBBG0.5/TBBG3/TBBG5 groups for brains with high *Q*^2^-values. Loading plots and S-plots revealed that the TBBG0.5/TBBG3/TBBG5 groups reversed most of the metabolic disturbances to normal levels in brains resulting from TMCAO. In addition, further testing was conducted to show the between-group differences in metabolites using univariate analysis. The results were visualized in the color-coded fold change tables (Table 2).

**Table 2:**
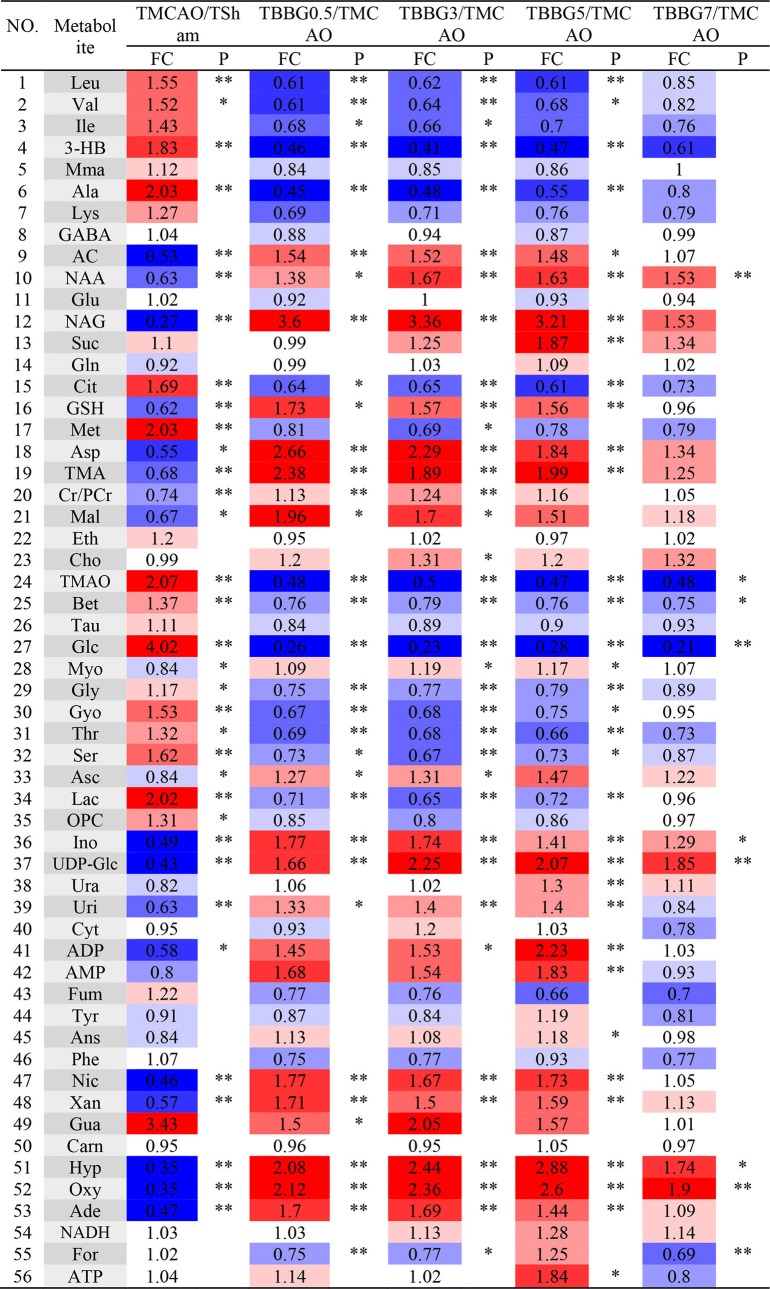
Potential marker metabolites and their fold changes^a^ among TMCAO treatment groups and the associated *P*-values^b^ in brain based on ^1^H NMR.

a *Color coded according to the fold change (FC), red represents higher and blue represents lower concentrations of metabolites. Color bar 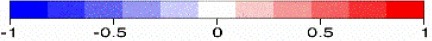
*.

b *P-values corrected by Benjamini–Hochberg methods were calculated based on a parametric Student's t-test or a nonparametric Mann–Whitney test*.

^*^p < 0.05,

^**^p < 0.01.

#### Shared and Unique Structure Analysis

**Figure 6:**
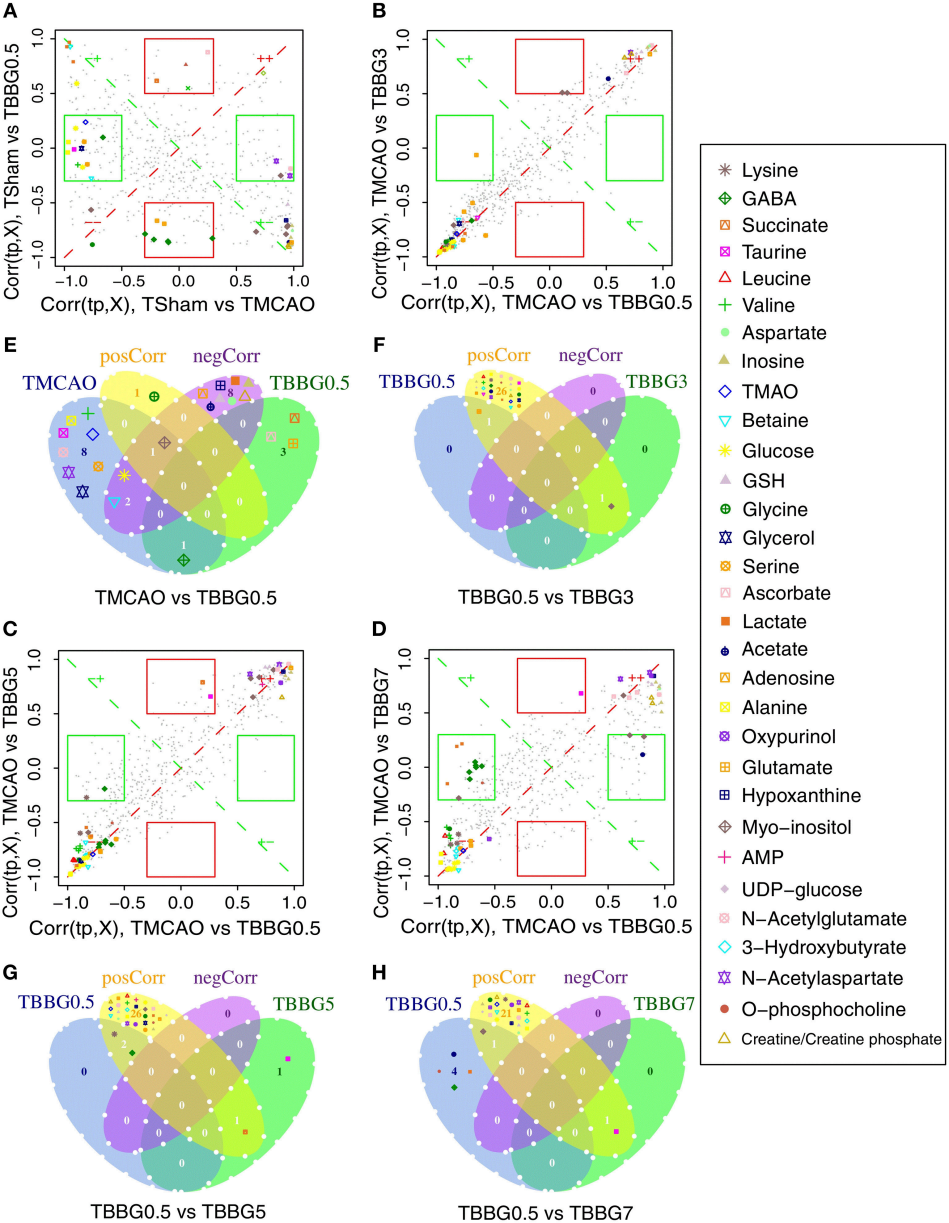
SUS plots and Venn plots of TBBG0.5 vs. tM, TBBG0.5 vs. TBBG3, TBBG0.5 vs. TBBG5, and TBBG0.5 vs. TBBG7 based on 1H NMR. The SUS plots **(A-D)** and Venn plots **(E-H)** of brain tissues show the shared and unique correlated metabolites. Shared effects are close to the diagonal lines (metabolites located on the red diagonal line are equally affected in both groups and located on the green diagonal line are negative correlated) while unique effects are located in the rectangles (red rectangle for TBBG0.5 and green rectangle for TBBG3/TBBG5/TBBG7). Venn plots illustrate the comparisons of metabolites among the TBBG0.5, TBBG3, TBBG5, and TBBG7 treatment groups.

The SUS analysis was of great value to investigate different mechanisms among BBG0.5, BBG3, BBG5, and BBG7 treatment groups. As it represented the earliest treatment, the TBBG0.5 group was compared to the TBBG3/TBBG5/TBBG7 groups to examine which metabolites were shared and unique (Figure 6). In the ^1^H NMR-based SUS plots of TBBG0.5 vs. TBBG3 and TBBG0.5 vs. TBBG5, 26 metabolites (e.g., leucine, valine, 3-hydroxybutyrate, etc.) close to the red diagonal line were positively correlated with TBBG0.5 and TBBG3/TBBG5; only one metabolite (taurine) was unique for TBBG5. However, four metabolites including acetate, o-phosphocholine, lactate, and GABA were all unique for TBBG0.5 when compared to TBBG7.

#### BBG Was Not Suitable for PMCAO Rats

According to an integrated analysis approach of BBG treatments in the PMCAO model, which included the metabolomics approach along with assessments of mortality, neurological defects, infarct volume, histopathology, immunohistochemistry, and biochemistry, no significant effects were observed, as indicated in the Supplementary Material (Figures S4B,D and S5–S8; Tables S3–S4).

## Discussion

Previous studies have demonstrated that HLJDD exerts obvious neuroprotective effects in stroke (Kondo et al., 2000; Xu et al., 2000). However, as a TCM, due to its complex chemical composition, quality control of HLJDD is difficult. Therefore, a refined formula derived from HLJDD with a clearly defined chemical composition and an equivalent or better efficacy is favorable. Berberine, baicalin, and geniposide, the three major components of HLJDD, have been proven to exert neuroprotective effects in ischemic stroke (Ahmed et al., 2015; Huang et al., 2017; Liang et al., 2017). According to previous works, neuroprotection of berberine and baicalin in I/R injury contribute to their anti-inflammatory and anti-oxidative effects as well as the improvement of energy metabolism, amino acid metabolism and nucleic acid metabolism (Tu et al., 2011a; Lv et al., 2017; Zhang et al., 2017a; Zhu et al., 2018). In addition, geniposide has been demonstrated to exert protective efficacy in I/R injury by alleviating energy metabolism, oxidative stress, and inflammatory responses (Wang et al., 2012). Therefore, a new formula BBG comprising of berberine, baicalin, and geniposide was developed, whose efficacy is responsible by all three mentioned components.

In this study, ^1^H NMR-based serum and brain metabolomics was coupled with various efficacy indexes including mortality rates, neurologic deficit scores, histopathology, infarct volumes, immunohistochemistry, and biochemical parameters to examine the neuroprotective properties of BBG in ischemic stroke. Metabolomics analysis including multivariate analysis, univariate analysis, and network analysis revealed that BBG is effective in the delayed treatment of cerebral ischemia-reperfusion injury, by alleviating oxidative stress and inflammation, improving energy supply and reversing disturbed amino acid and nucleic acid metabolisms. A schematic diagram of the disturbed metabolic pathways is depicted in Figure 9. Our results show that BBG treatment is not suitable for PMCAO treatment but good for TMCAO treatment by reducing infarct volumes and ameliorating neurological deficits, even extending up to 5 h after reperfusion (TBBG0.5, TBBG3, and TBBG5 groups). Therefore, the effective therapeutic time window for BBG is 5 h after reperfusion. Treatment beyond this time window (TBBG7) was ineffective. These results suggest that BBG is competent in the treatment of TMCAO in rodents with a favorable 5-h therapeutic window, which is of great clinical importance (Fisher et al., 2009). BBG showed therapeutic effects comparable to HLJDD, and better than each individual compound in ischemic stroke rats (Figure S3). These results suggest that BBG could be an effective alternative to HLJDD.

### Energy Metabolism

Compared with Sham rats, the levels of glucose, citrate, 3-HB, lactate, and fumarate were obviously increased, while levels of ADP and creatine/phosphocreatine were significantly decreased in TMCAO rats. During ischemic stroke, cerebrovascular occlusion cuts off blood supply, resulting first in depletion of oxygen and glucose in the brain, and later in disturbances of energy metabolism (Villa et al., 2009). As indicated by the increased levels of citrate and fumarate, the Krebs cycle was markedly blocked in TMCAO rats. The Krebs cycle is the major energy source of cells, and thus its blockage leads to the depletion of brain energy, as indicated by the significant decreases of ADP, AMP, adenosine, inosine, hypoxanthine, and xanthine (Barsotti and Ipata, 2004). As a result of this, the energy metabolism in the TMCAO rat brains shifted to anaerobic glycolysis to rescue depleted energy, as indicated by the significantly increased level of lactate. However, anaerobic glycolysis was not sufficient to completely satisfy brain energy requirements, which called for activation of other energy-yielding metabolic pathways including those involving ketone bodies and the creatine/phosphocreatine (Cr/PCr) system. For example, the ketone body 3-HB could serve as fuel to participate in energy generation when the brain was undergoing starvation (Newman and Verdin, 2014). In this study, 3-HB levels were significantly decreased in serum but increased in ischemic brains, suggesting that 3-HB was being transferred from the blood to the brain to supply extra energy to brain cells, which are extremely sensitive to low glucose levels and highly prone to death due to energy deficiency. The Cr/PCr system, through the ATP-producing creatine kinase (CK) reaction, played an important role in sustaining ATP levels and attenuating ischemic injury (Ma et al., 2010; Wallimann et al., 2011). Decreased levels of Cr/PCr in the ischemic brain indicated an accelerated consumption of Cr/PCr as an emergency energy-producing protective mechanism.

Here, BBG treatment within 5 h markedly alleviated the malfunction of the energy metabolism in TMCAO rats, as indicated by the significantly decreased levels of 3-HB, citrate, fumarate, glucose, and lactate, along with the increased levels of ADP and creatine/phosphocreatine. According to SUS analysis, lactate is a unique metabolite for TBBG7 compared to TBBG0.5 (Figures 6D,H). As a marker of anaerobic glycolysis, increased lactate and activated glycolysis could be used as markers for poor prognostics of stroke (Ide et al., 1969).

### Oxidative Stress

Reperfusion after ischemia usually brings large amounts of oxygen to the neurons. Consequently, ROS are immediately formed during the reperfusion phase by the combination of excessive oxygen with electrons leaking from the damaged mitochondrial electron transport chain (ETC) in the dying cells. This leads to secondary injury including large-scale degradation of DNA and proteins and peroxidation of cellular lipids (Liu et al., 2010). In our study, the oxidative status was determined by measuring a series of biochemical parameters including SOD, GSH, GR, MDA, CAT, NO, GSSG, and GSH-Px. In a separate study of the TMCAO model, a significantly increased level of MDA suggested severe lipid peroxidation in the brain cells (Niedernhofer et al., 2003). In addition, excessive NO could react with ROS to produce neurotoxic peroxynitrite, which is abundant in cells injured by cerebral ischemia-reperfusion (Pacher et al., 2007). SOD and CAT, two famous antioxidant enzymes, could cooperate to promote a disproportionation reaction and turn O^2−^ and H_2_O_2_ into H_2_O to relieve oxidative stress (Pinho et al., 2006). In the GSH redox system, GSH could be oxidized to GSSG under the catalysis of GSH-Px, and GSSG could be reduced to GSH by GR to decrease equivalents derived from NADPH (Zitka et al., 2012). Here, the clearly increased levels of GSSG and decreased levels of SOD, CAT, GSH, GR, and GSH-Px revealed excessive ROS production and severe oxidative status in TMCAO rats. ROS then induced degradation of phospholipids in cellular membranes, as revealed by an increased level of glycerol, a hydrolysate of membrane phospholipid (Hillered et al., 1998). ROS could also attack proteins and lead to their degradation into amino acids (Batch et al., 2014). Therefore, levels of amino acids, such as BCAAs (leucine, isoleucine, and valine) were significantly increased in TMCAO rats.

Activities of SOD, CAT, and GSH in 3 h treatments were similar to those in 0.5 h treatments (*P* > 0.05) and higher than what was observed from the 5 and 7 h treatment groups (*P* < 0.05), suggesting that during reperfusion, the anti-oxidative capabilities of BBG started from a 3-h incubation period followed by an accelerated loss phase. Treatment within a 3-h incubation period led to a maximal rescue of the antioxidant enzyme system in the brain. Levels of MDA, NO and GSSG in the 5 h treatment was similar to those in 3 h treatments (*P* > 0.05) and lower than those in 7 h treatments (*P* < 0.05), indicating that damage to the brain was in a stable phase (3–5 h) and then went into an accelerated phase. BBG treatment at this stable phase led to the most effective inhibition of brain damage exacerbation mechanisms. Overall, the whole oxidative status could be significantly ameliorated by BBG treatment within 5 h.

**Figure 7:**
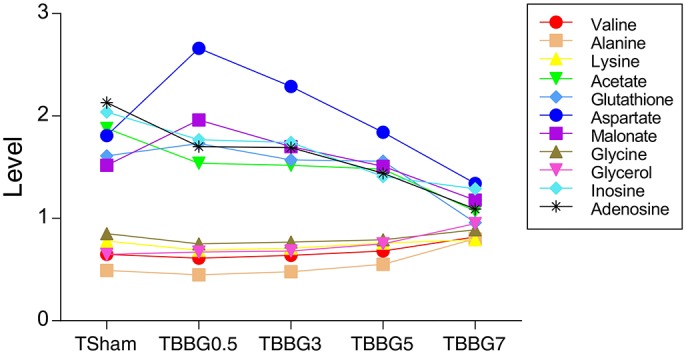
Progressively increased and decreased levels of metabolites in TMCAO treatment groups and Sham group. Progressively increased metabolites include valine, alanine, lysine, glycerol, and glycine. Progressively decreased metabolites include aspartate, glutathione, inosine, adenosine, acetate and malonate.

**Figure 8:**
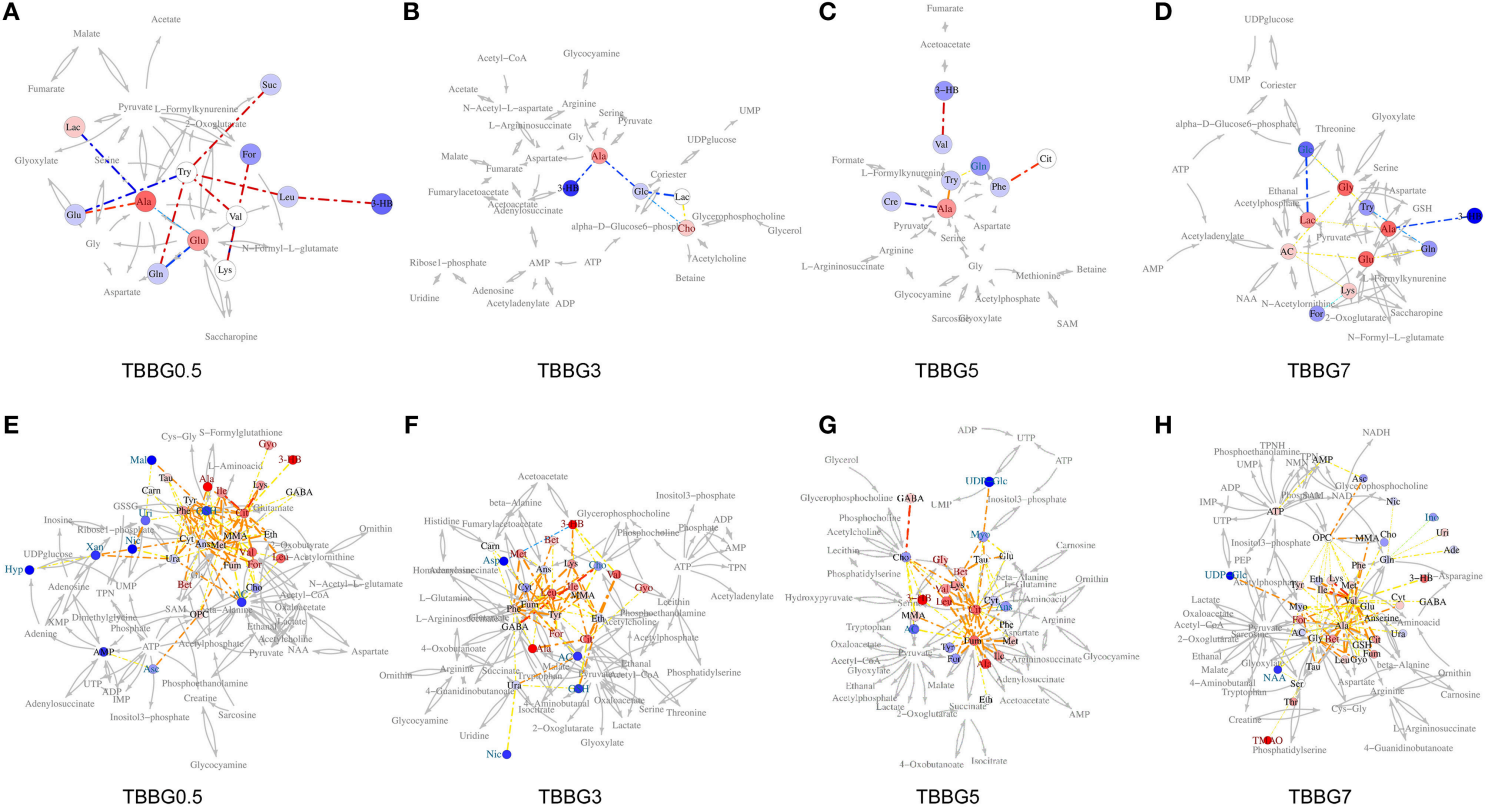
Correlation network of BBG treatment in TMCAO rats. Network analysis of TBBG0.5, TBBG3, TBBG5, and TBBG7 treatment groups in serum **(A–D)** and ischemic brain **(E–H)**. The networks are constructed by connecting metabolites with dotted lines colored warm or cool to represent positive or negative correlation. The width represents absolute values of coefficients. The warm or cool color of metabolites represents obvious increased or decreased levels in each treatment groups.

It is worth mentioning that levels of glycerol and valine at 24 h after stroke showed a steady increase from 0.5 to 7 h treatments (Figure 7). Therefore, the two metabolites could be used to assess the severity of stroke and the efficacy of its treatments. In the correlation networks (Figures 8E–H), valine was only marginal in the networks for the treatments within 5 h but in the center of network for the treatment at 7 h. In addition, it showed a strong correlation with many metabolites including 3-HB, citrate, fumarate and so on, revealing a turning point at 7 h.

**Figure 9:**
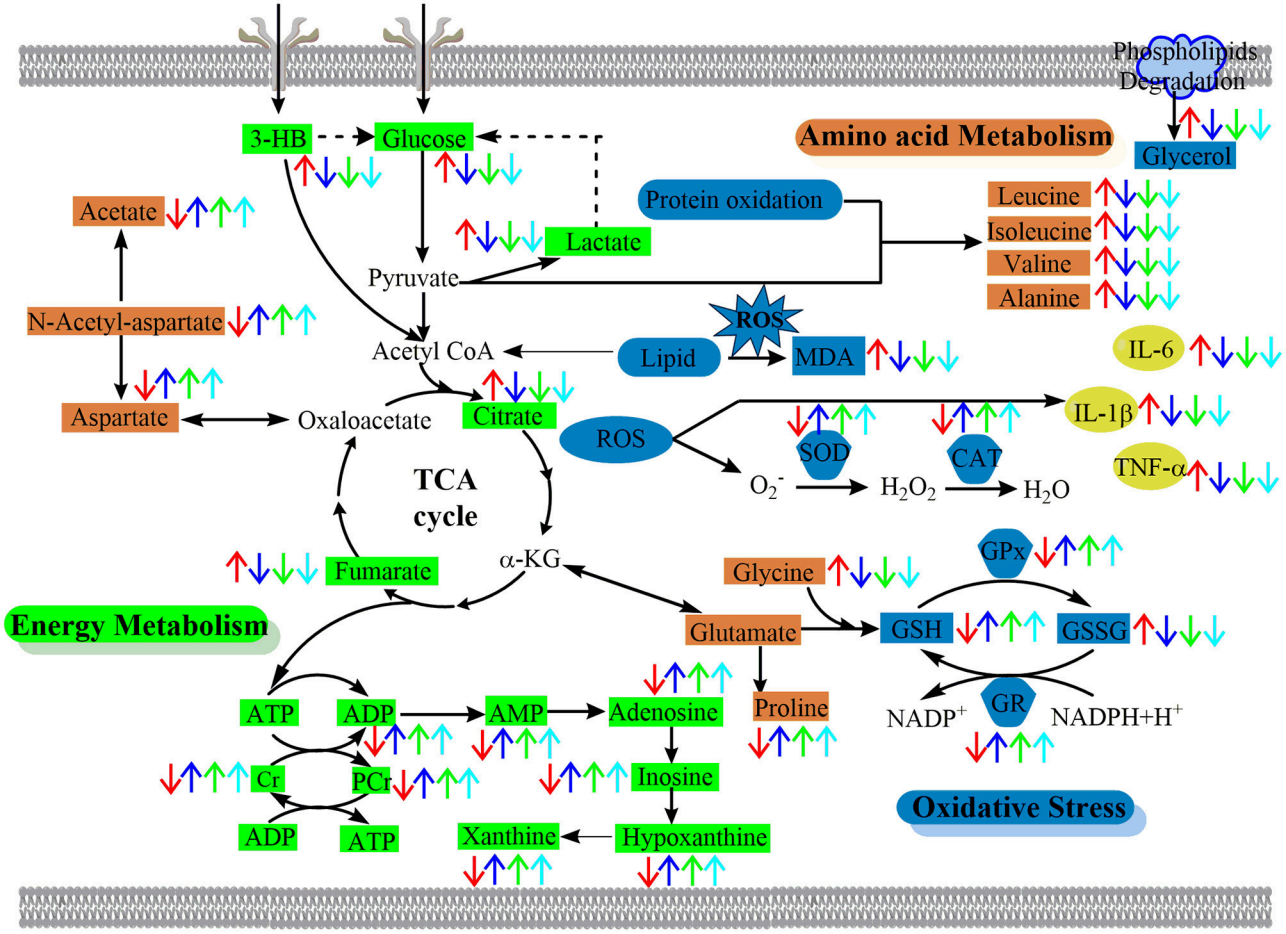
The schematic diagram of the perturbed pathways according to ^1^H NMR analysis of ischemic brain. BBG exerted its neuroprotective effect against cerebral ischemia-reperfusion injury by reversing oxidative stress, alleviating inflammation and improving the disturbed energy, nucleic acid, and amino acids metabolism to the normal status. Arrows (“↑↓”) in different colors represent the significant increases or decreases of metabolites in the TMCAO (red arrows), TBBG0.5 (deep blue arrows), TBBG3 (green arrows), and TBBG5 (cyan arrows) groups, respectively. BCAAs, branched-chain amino acids; α-KG, α-ketoglutarate; TCA cycle, tricarboxylic acid cycle.

### Amino Acid Metabolism

Aspartate and N-acetyl-aspartate (NAA) were significantly decreased while glycine, taurine, and alanine were markedly increased in the brain of the TMCAO group when compared to the Sham group. Aspartate, a well-known excitatory amino acid (Zhang et al., 2017b), was significantly decreased in TMCAO rats. Neurotoxicity induced by aspartate plays a key role in hypoxic-ischemic neuronal death. Here, the decreased levels of aspartate may be attributed to the Krebs cycle being inhibited. To compensate for the inefficient function of the Krebs cycle, by replenishing its intermediates, aspartate was converted to oxaloacetate and then to citrate. Another explanation for the low levels of aspartate may be that the decreased levels of NAA (precursor of aspartate) contributed to insufficient production of aspartate. NAA, one of the most concentrated metabolites in the brain, is used as a marker for neuronal completeness because its synthetase is solely localized in the mitochondria membrane of the neurons (Moffett et al., 2007; Silachev et al., 2015). Since it is hydrolyzed to aspartate and acetate by the action of aspartoacylase II, NAA is beneficial for lipid synthesis by releasing acetate. Therefore, decreased levels of NAA also resulted in a decrease of acetate levels and reflected severe neuronal damage and loss, which was consistent with the histopathological assessments. In contrast, inhibitory amino acids such as glycine and taurine, whose levels were significantly increased, were released after TMCAO and served to neutralize the toxicity caused by excitatory amino acids (Zhang et al., 2017b). Besides, during ischemia-reperfusion injury, alanine was also increased due to the flux of pyruvate through the TCA. As previously reported, it is possible that the increased levels of alanine would damage the mitochondrial respiratory chain by inhibiting mitochondrial Complex I-III in the ischemic brains of TMCAO rats (Virginia Cielo et al., 2002).

The increased levels of glycine, taurine, and alanine and decreased levels of aspartate and NAA were significantly reversed even if the therapy was delayed up to 5 h after reperfusion. Importantly, the levels of alanine and glycine at 24 h after stroke showed a steady increase and that of aspartate showed a steady decrease from 0.5 to 7 h treatments (Figure 7), suggesting the ability of BBG to improve amino acid metabolism declined over time. Alanine showed strong correlations to other metabolites in both serum metabolomics (Figures 8A–D) and brain metabolomics (Figures 8E–H). According to network analysis of brain metabolomics (Figures 8E–H), alanine located in the center of 7 h treatment but in the edge from 0.5 to 5 h treatment; in addition, it linked to many other amino acids such as leucine, isoleucine, valine and so on, suggesting that alanine metabolism was severely disrupted when the treatment was delayed to 7 h after stroke, which strongly correlated with protein degradation.

### Inflammation Response

Inflammation response is a later event associated with neutrophil infiltration and activation of glial cells in the ischemic penumbra several hours after reperfusion (Iadecola and Anrather, 2012). Previous studies have reported that the pro-inflammatory cytokines were increased in the TMCAO (Oto et al., 2008; Tu et al., 2011a). In this study, we demonstrated that cerebral ischemia-reperfusion injury caused elevated levels of TNF-α, IL-1β, and IL-6 in the serum. In addition, as a marker for astrocytes, increased levels of GFAP indicated the activation of astrocytes in the brain (Eng and Ghirnikar, 2010; Figure 1E). In the ischemic stroke, astrocytes are involved in the cross-talk between brain immune cells by supplying a source of pro-inflammatory cytokines and eventually activating infiltrating lymphocytes. Indeed, astrocytes can produce many factors such as IL-1β and IL-6, to generate potent pro-inflammatory functions (Gu et al., 2013). Besides, myo-inositol is a glial marker, whose decreased level in TMCAO suggested that the ionic balance was severely disrupted and eventually resulted in brain edema (Haris et al., 2011).

Levels of TNF-α, IL-1β, IL-6, and GFAP were significantly decreased when BBG was applied within 5 h. Our results showed that levels of TNF-α, IL-1β, and IL-6 in the 3 h treatment were similar to those in the 5 h treatment (*P* > 0.05) and much higher than those in the 0.5 h treatment (*P* < 0.05). This suggests that inflammatory responses were quick, starting only a few minutes after reperfusion and then reaching a plateau from 3 to 5 h. Moreover, the levels of TNF-α, IL-1β, and IL-6 in the 0.5 h treatment were the same as their levels in the Sham group (*P* > 0.05). This result indicates that early treatment of BBG (0.5 h) can maximally block the inflammatory cascade initiated at the onset.

### Nucleic Acid Metabolism

The levels of inosine, adenosine, xanthine, and hypoxanthine were considerably decreased in the TMCAO group. ROS produced in cerebral ischemia-reperfusion could attack DNA, leading to its breaking. Inosine is an endogenous nucleoside metabolized from adenosine (Hagberg et al., 2010). Inosine could be further metabolized into hypoxanthine, and the latter was metabolized into xanthine. Finally, uric acid, a potent free-radical scavenger, was generated from xanthine. Great consumption of nucleic acids, as indicated by decreased levels of inosine, adenosine, xanthine, and hypoxanthine, were used to synthesize DNA again, to perform DNA repair.

### Final Remarks

Previous studies have reported that HLJDD and its active components could improve ischemic stroke (Xu et al., 2000; Zhang et al., 2017a). However, most of them focused on the neuroprotection of the whole formula or its single component (Tu et al., 2011b; Ahmed et al., 2015; Huang et al., 2017; Zhu et al., 2018). In addition, the therapeutic window and suited stroke types of HLJDD or its components have not been clarified by previous studies as of yet. All these issues were addressed for the first time, by the metabolomics approach used in this study. The refined drug BBG can achieve efficacy comparable to HLJDD in cerebral I/R injury but is ineffective in continuous ischemia injury. BBG exerts the efficacy within a 5-h therapeutic window by reversing the disturbed energy metabolism, amino acid metabolism, nucleic acid metabolism, oxidative stress, and inflammation. All these findings suggest that BBG can be a good alternative to HLJDD, and supports its further development as a drug for stroke treatment.

## Ethics Statement

This study was carried out in accordance with the recommendations of Animal Ethics Committee of China Pharmaceutical University. The protocol was approved by Animal Ethics Committee of China Pharmaceutical University.

## Author Contributions

JW, MY, and LK conceived the experiments and helped to coordinate support and funding. XF performed the research and drafted the manuscript. SL, YL, and DX participated in the experiments. JW analyzed the data and edited the paper. All authors read and approved the final manuscript.

### Conflict of Interest Statement

The authors declare that the research was conducted in the absence of any commercial or financial relationships that could be construed as a potential conflict of interest.

## References

[B1] Ahmed T. Gilani A. U. Abdollahi M. Daglia M. Nabavi S. F. Nabavi S. M. (2015). Berberine and neurodegeneration: a review of literature. Pharmacol. Rep. 67, 970–979. 10.1016/j.pharep.2015.03.002 26398393

[B2] Baker M. (2011). Metabolomics: from small molecules to big ideas. Nat. Methods 8:117 10.1038/nmeth0211-117

[B3] Barsotti C. Ipata P. L. (2004). Metabolic regulation of ATP breakdown and of adenosine production in rat brain extracts. Int. J. Biochem. Cell B 36:2214. 10.1016/j.biocel.2004.04.015 15313467

[B4] Batch B. C. Hyland K. Svetkey L. P. (2014). Branch chain amino acids: biomarkers of health and disease. Curr. Opin. Clin. Nutr. 17, 86–89. 10.1097/MCO.0000000000000010 24310057

[B5] Bederson J. B. Pitts L. H. Germano S. M. Nishimura M. C. Davis R. L. Bartkowski H. M. (1986). Evaluation of 2,3,5-triphenyltetrazolium chloride as a stain for detection and quantification of experimental cerebral infarction in rats. Stroke 17:1304. 10.1161/01.STR.17.6.1304 2433817

[B6] De Meyer T. Sinnaeve D. Van Gasse B. Tsiporkova E. Rietzschel E. R. De Buyzere M. L.. (2008). NMR-based characterization of metabolic alterations in hypertension using an adaptive, intelligent binning algorithm. Anal. Chem. 80, 3783–3790. 10.1021/ac7025964 18419139

[B7] Donnan G. A. Fisher M. Macleod M. Davis S. M. (2008). Stroke. Lancet 371, 1612–1623. 10.1016/S0140-6736(08)60694-7 18468545

[B8] Eng L. F. Ghirnikar R. S. (2010). GFAP and astrogliosis. Brain Pathol. 4, 229–237. 10.1111/j.1750-3639.1994.tb00838.x 7952264

[B9] Fisher M. Feuerstein G. Howells D. W. Hurn P. D. Kent T. A. Savitz S. I.. (2009). Update of the stroke therapy academic industry roundtable preclinical recommendations. Stroke 40, 2244–2250. 10.1161/STROKEAHA.108.541128 19246690 PMC2888275

[B10] Fisher M. Hanley D. F. Howard G. Jauch E. C. Warach S. Group S. (2007). Recommendations from the STAIR V meeting on acute stroke trials, technology and outcomes. Stroke 38, 245–248. 10.1161/01.STR.0000255951.37434.aa 17204668

[B11] Gu L. Huang B. Shen W. Gao L. Ding Z. Wu H.. (2013). Early activation of nSMase2/ceramide pathway in astrocytes is involved in ischemia-associated neuronal damage via inflammation in rat hippocampi. J Neuroinflamm 10:879. 10.1186/1742-2094-10-109 24007266 PMC3844623

[B12] Gupta S. Sharma U. Jagannathan N. R. Gupta Y. K. (2017). Neuroprotective effect of lercanidipine in middle cerebral artery occlusion model of stroke in rats. Exp. Neurol. 288, 25–37. 10.1016/j.expneurol.2016.10.014 27794423

[B13] Hagberg H. Andersson P. Lacarewicz J. Jacobson I. Butcher S. Sandberg M. (2010). Extracellular adenosine, inosine, hypoxanthine, and xanthine in relation to tissue nucleotides and purines in rat striatum during transient ischemia. J. Neurochem. 49, 227–231. 10.1111/j.1471-4159.1987.tb03419.x 3585332

[B14] Haris M. Cai K. Singh A. Hariharan H. Reddy R. (2011). *In vivo* mapping of brain myo-inositol. Neuroimage 54, 2079–2085. 10.1016/j.neuroimage.2010.10.017 20951217 PMC3013615

[B15] Hillered L. Valtysson J. Enblad P. Persson L. (1998). Interstitial glycerol as a marker for membrane phospholipid degradation in the acutely injured human brain. J. Neurol. Neurosurg. Psychiatry 64, 486–491. 10.1136/jnnp.64.4.486 9576540 PMC2170060

[B16] Huang B. Chen P. Huang L. Li S. Zhu R. Sheng T.. (2017). Geniposide attenuates post-ischaemic neurovascular damage via GluN2A/AKT/ERK-dependent mechanism. Cell. Physiol. Biochem. 43, 705–716. 10.1159/000480657 28957809

[B17] Iadecola C. Anrather J. (2012). The immunology of stroke: from mechanisms to translation. Nat. Med. 17, 796–808. 10.1038/nm.2399 21738161 PMC3137275

[B18] Ide T. Steinke J. Cahill G. F. Jr. (1969). Metabolic interactions of glucose, lactate, and beta-hydroxybutyrate in rat brain slices. Am. J. Physiol. 217, 784–792. 10.1152/ajplegacy.1969.217.3.784 5807702

[B19] Kondo Y. Kondo F. Asanuma M. Tanaka K.-I. Ogawa N. (2000). Protective effect of oren-gedoku-to against induction of neuronal death by transient cerebral ischemia in the C57BL/6 mouse. Neurochem. Res. 25, 205–209. 10.1023/A:1007515318434 10786703

[B20] Liang W. Huang X. Chen W. (2017). The effects of baicalin and baicalein on cerebral ischemia: a review. Aging Dis. 8, 850–867. 10.14336/AD.2017.0829 29344420 PMC5758355

[B21] Liu Y. Fiskum G. Schubert D. (2010). Generation of reactive oxygen species by the mitochondrial electron transport chain. J. Neurochem. 80, 780–787. 10.1046/j.0022-3042.2002.00744.x 11948241

[B22] Longa E. Z. Weinstein P. R. Carlson S. Cummins R. (1989). Reversible middle cerebral artery occlusion without craniectomy in rats. Stroke 20:84. 10.1161/01.STR.20.1.84 2643202

[B23] Lozano R. Naghavi M. Foreman K. Lim S. Shibuya K. Aboyans V.. (2012). Global and regional mortality from 235 causes of death for 20 age groups in 1990 and 2010: a systematic analysis for the Global Burden of Disease Study 2010. Lancet 380, 2095–2128. 10.1016/S0140-6736(12)61728-0 23245604 PMC10790329

[B24] Lv Y. Wang J. Xu D. Liao S. Li P. Zhang Q.. (2017). Comparative study of single/combination use of Huang-Lian-Jie-Du decoction and berberine on their protection on sepsis induced acute liver injury by NMR metabolic profiling. J. Pharmaceut. Biomed. 145, 794–804. 10.1016/j.jpba.2017.07.062 28822346

[B25] Ma C. Bi K. Zhang M. Su D. Fan X. Ji W.. (2010). Metabonomic study of biochemical changes in the urine of Morning Glory Seed treated rat. J. Pharmaceut. Biomed. 53, 559–566. 10.1016/j.jpba.2010.03.034 20403675

[B26] Matucz E. Móricz K. Gigler G. Benedek A. Barkóczy J. Lévay G.. (2006). Therapeutic time window of neuroprotection by non-competitive AMPA antagonists in transient and permanent focal cerebral ischemia in rats. Brain Res. 1123, 60–67. 10.1016/j.brainres.2006.09.043 17064671

[B27] Moffett J. R. Ross B. Arun P. Madhavarao C. N. Namboodiri A. M. (2007). N-Acetylaspartate in the CNS: from neurodiagnostics to neurobiology. Prog. Neurobiol. 81, 89–131. 10.1016/j.pneurobio.2006.12.003 17275978 PMC1919520

[B28] Mozaffarian D. Benjamin E. J. Go A. S. Arnett D. K. Blaha M. J. Cushman M. (2016). Executive summary: heart disease and stroke statistics-2016 update: a report from the American Heart Association. Circulation 127, 143–152. 10.1161/CIR.0000000000000366 23283859

[B29] Newman J. C. Verdin E. (2014). Ketone bodies as signaling metabolites. Trends Endocrin. Met. 25, 42–52. 10.1016/j.tem.2013.09.002 24140022 PMC4176946

[B30] Niedernhofer L. J. Daniels J. S. Rouzer C. A. Greene R. E. Marnett L. J. (2003). Malondialdehyde, a product of lipid peroxidation, is mutagenic in human cells. J. Biol. Chem. 278:31426. 10.1074/jbc.M212549200 12775726

[B31] Nordström A. Lewensohn R. (2010). Metabolomics: moving to the clinic. J. Neuroimmune Pharm. 5, 4–17. 10.1007/s11481-009-9156-4 19399626

[B32] O'donnell M. J. Chin S. L. Rangarajan S. Xavier D. Liu L. Zhang H.. (2016). Global and regional effects of potentially modifiable risk factors associated with acute stroke in 32 countries (INTERSTROKE): a case-control study. Lancet 388, 761–775. 10.1016/S0140-6736(16)30506-2 27431356

[B33] Oto J. Suzue A. Inui D. Fukuta Y. Hosotsubo K. Torii M.. (2008). Plasma proinflammatory and anti-inflammatory cytokine and catecholamine concentrations as predictors of neurological outcome in acute stroke patients. J. Anesth. 22, 207–212. 10.1007/s00540-008-0639-x 18685925

[B34] Pacher P. Beckman J. S. Liaudet L. (2007). Nitric oxide and peroxynitrite in health and disease. Physiol. Rev. 87, 315–424. 10.1152/physrev.00029.2006 17237348 PMC2248324

[B35] Pinho R. A. Andrades M. E. Oliveira M. R. Pirola A. C. Zago M. S. Silveira P. C.. (2006). Imbalance in SOD/CAT activities in rat skeletal muscles submitted to treadmill training exercise. Cell Bio. Int. 30, 848–853. 10.1016/j.cellbi.2006.03.011 17011801

[B36] Silachev D. N. Gulyaev M. V. Zorova L. D. Khailova L. S. Gubsky L. V. Pirogov Y. A.. (2015). Magnetic resonance spectroscopy of the ischemic brain under lithium treatment. Link to mitochondrial disorders under stroke. Chem. Biol. Interact. 237, 175–182. 10.1016/j.cbi.2015.06.012 26079057

[B37] Tu X. K. Yang W. Z. Liang R. S. Shi S. S. Chen J. P. Chen C. M.. (2011a). Effect of baicalin on matrix metalloproteinase-9 expression and blood-brain barrier permeability following focal cerebral ischemia in rats. Neurochem. Res. 36, 2022–2028. 10.1007/s11064-011-0526-y 21678122

[B38] Tu X. K. Yang W. Z. Shi S. S. Chen Y. Wang C. H. Chen C. M.. (2011b). Baicalin inhibits TLR2/4 signaling pathway in rat brain following permanent cerebral ischemia. Inflammation 34, 463–470. 10.1007/s10753-010-9254-8 20859668

[B39] Villa R. F. Gorini A. Hoyer S. (2009). Effect of ageing and ischemia on enzymatic activities linked to Krebs' cycle, electron transfer chain, glutamate and aminoacids metabolism of free and intrasynaptic mitochondria of cerebral cortex. Neurochem. Res. 34, 2102–2116. 10.1007/s11064-009-0004-y 19495970

[B40] Virginia Cielo R. Luciane Rosa F. Carlos Severo D.F. Wyse A.T.D.S. Moacir W. Wannmacher C.M.D. (2002). Inhibition of the mitochondrial respiratory chain by alanine in rat cerebral cortex. Metab. Brain. Dis. 17, 123–130. 10.1023/A:1019973719399 12322782

[B41] Wallimann T. Tokarska-Schlattner M. Schlattner U. (2011). The creatine kinase system and pleiotropic effects of creatine. Amino Acids 40, 1271–1296. 10.1007/s00726-011-0877-3 21448658 PMC3080659

[B42] Wang J. Hou J. Zhang P. Li D. Zhang C. Liu J. (2012). Geniposide reduces inflammatory responses of oxygen-glucose deprived rat microglial cells via inhibition of the TLR4 signaling pathway. Neurochem. Res. 37, 2235–2248. 10.1007/s11064-012-0852-8 22869019

[B43] Wang P. R. Wang J. S. Yang M. H. Kong L. Y. (2014). Neuroprotective effects of Huang-Lian-Jie-Du-Decoction on ischemic stroke rats revealed by ^1^H NMR metabolomics approach. J. Pharmaceut. Biomed. 88, 106–116. 10.1016/j.jpba.2013.08.025 24051274

[B44] Xu J. Murakami Y. Matsumoto K. Tohda M. Watanabe H. Zhang S.. (2000). Protective effect of Oren-gedoku-to (Huang-Lian-Jie-Du-Tang) against impairment of learning and memory induced by transient cerebral ischemia in mice. J. Ethnopharmacol. 73, 405–413. 10.1016/S0378-8741(00)00303-2 11090993

[B45] Xu Z. Croslan D. R. Harris A. E. Ford G. D. Ford B. D. (2006). Extended therapeutic window and functional recovery after intraarterial administration of neuregulin-1 after focal ischemic stroke. J. Cereb. Blood Flow Metab. 26, 527–535. 10.1038/sj.jcbfm.9600212 16136057

[B46] Yang Q. Fang W. Lv P. Geng X. Li Y. Sha L. (2012). Therapeutic neuroprotective effects of XQ-1H in a rat model of permanent focal cerebral ischemia. Pharmacology 89, 1–6. 10.1159/000334625 22178991

[B47] Zeynalov E. Jones S. M. Elliott J. P. (2017). Therapeutic time window for conivaptan treatment against stroke-evoked brain edema and blood-brain barrier disruption in mice. PLoS ONE 12:e0183985. 10.1371/journal.pone.0183985 28854286 PMC5576654

[B48] Zhang Q. Fu X. Wang J. Yang M. Kong L. (2017a). Treatment effects of ischemic stroke by Berberine, Baicalin, and Jasminoidin from Huang-Lian-Jie-Du-Decoction (HLJDD) explored by an integrated metabolomics approach. Oxid. Med. Cell. Longev. 2017:9848594. 10.1155/2017/9848594 28894512 PMC5574319

[B49] Zhang Q. Wang J. Liao S. Li P. Xu D. Lv Y.. (2017b). Optimization of Huang-Lian-Jie-Du-Decoction for ischemic stroke treatment and mechanistic study by metabolomic profiling and network analysis. Front. Pharmacol. 8:165. 10.3389/fphar.2017.00165 28400733 PMC5368223

[B50] Zhang Y. Wang L. Li J. Wang X. L. (2006). 2-(1-Hydroxypentyl)-benzoate increases cerebral blood flow and reduces infarct volume in rats model of transient focal cerebral ischemia. J. Pharmacol. Exp. Ther. 317, 973–979. 10.1124/jpet.105.098517 16527903

[B51] Zhu B. Cao H. Sun L. Li B. Guo L. Duan J.. (2018). Metabolomics-based mechanisms exploration of Huang-Lian Jie-Du decoction on cerebral ischemia via UPLC-Q-TOF/MS analysis on rat serum. J. Ethnopharmacol. 216, 147–156. 10.1016/j.jep.2018.01.015 29360497

[B52] Zhu H. Qian Z. He F. Liu M. Pan L. Zhang Q.. (2013). Novel pharmacokinetic studies of the Chinese formula Huang-Lian-Jie-Du-Tang in MCAO rats. Phytomedicine 20, 767–774. 10.1016/j.phymed.2012.11.012 23628154

[B53] Zitka O. Skalickova S. Gumulec J. Masarik M. Adam V. Hubalek J.. (2012). Redox status expressed as GSH:GSSG ratio as a marker for oxidative stress in paediatric tumour patients. Oncol. Lett. 4:1247. 10.3892/ol.2012.931 23205122 PMC3506742

