# Supplementary material for: 1H NMR-Based Metabolomics Reveals Refined-Huang-Lian-Jie-Du-Decoction (BBG) as a Potential Ischemic Stroke Treatment Drug With Efficacy and a Favorable Therapeutic Window
Source: Front Pharmacol. 2019 Apr 12;10:337. doi: 10.3389/fphar.2019.00337 (PMC6474285; doi:10.3389/fphar.2019.00337)
Supplement: Supplementary file 1 [file Data_Sheet_1.docx]

**Supplementary Material**

^1^H NMR-Based Metabolomics Reveals Refined-Huang-Lian-Jie-Du-Decoction (BBG) as a Potential Ischemic Stroke Treatment Drug with Efficacy and a Favorable Therapeutic Window

**Xiaowei Fu^1^, Junsong Wang^2 *^****, Shanting Liao^1^, Yan Lv^1^, Dingqiao Xu^1^, Minghua Yang^1^, Lingyi Kong^1 *^**

^1^ Jiangsu Key Laboratory of Bioactive Natural Product Research and State Key Laboratory of Natural Medicines, School of Traditional Chinese Pharmacy, China Pharmaceutical University, 24 Tong Jia Xiang, Nanjing, 210009, P.R. China

^2^ Center for Molecular Metabolism, School of Environmental and Biological Engineering, Nanjing University of Science and Technology, 200 Xiao Ling Wei Street, Nanjing, 210094, P.R. China

**^*^** **Corresponding authors:**

Lingyi Kong

E-mail: [cpu_lykong@126.com](mailto:cpu_lykong@126.com)

Tel/Fax: +86 25 8327 1405

Junsong Wang

E-mail: wang.junsong@gmail.com

Tel: +86 25 8431 5512

**Supplementary Results**

**BBG Could Not Reduce Mortality, Neurological Defect or Cerebral Infarction, or Improve Histopathological Injury, Anti-oxidative and Anti-inflammatory Capacities** **of PMCAO Rats**

For continuous ischemia, neither PBBG2 nor PBBG4 had an evident reduction in mortality, neurological scores. or infarct volumes (**Figure S4 A** and **C**). Moreover, pathological abnormalities were still remarkably observed in PBBG2 and PBBG4 treatment group (**Figure S4 B)**. Besides, biochemical indexes were still severe with the BBG treatment in PMCAO rats (**Figure S4 D)**.

**BBG Performed Poorly in The Amelioration of Pathological Metabolic Disturbances in PMCAO**

**Serum Metabolomic Analysis**

In the OPLS-DA score plots from the PMCAO, PBBG2, PBBG4 and Sham groups (**Figure S5 A)**, the integral metabolic patterns of PBBG2 and PBBG4 groups were far away from the Sham group and close to PMCAO group. The results indicated that BBG treatment within 2 h or 4 h after onset of PMCAO exerted few therapeutic effects.

A further exploration was performed to identify if any metabolite in the serum of PMCAO rats could be improved by BBG **(Figure S5 B)**. In the score plot, the PMCAO group and Sham group showed clear separation with good fit (R^2^Y = 0.96, Q^2^Y = 0.88, **Figure S3 b**), suggesting an excellent PMCAO model. The color-coded loading plots and S-plots showed a large number of disordered metabolites in the PMCAO group as compared with the Sham group. Further exploration was conducted to show between-group difference of metabolites using univariate analysis as visualized in the colored table **(Table S3)**. Among these changed metabolites, only 3-hydroxybutyrate, methylmalonate, alanine and glycine could be reversed in PBBG2 or PBBG4 treatment groups.

**Brain Metabolomic Analysis**

The metabolomic data of brain samples from PMCAO, PBBG2, PBBG4 and Sham groups were explored by OPLS-DA model to uncover if BBG is effective in continuous ischemic hemicerebrum. The score plots showed that the PMCAO group and Sham group were the furthest away **(Figure S6 A)** with PBBG2 and PBBG4 treatment groups far away from the Sham group and close to the PMCAO group, suggesting disturbed metabolic patterns in continuous ischemia injury could not be improved by BBG.

To examine metabolic mechanisms in the continuous ischemic brains and identify metabolites related to the slight therapeutic effects, the Sham, PMCAO, t2H, and t4H groups were explored by OPLS-DA models, individually **(Figure S6 B)**. Compared PMCAO group with Sham group and PBBG2/PBBG4 group, score plots for brain exhibited a complete separation of the Sham and PMCAO with a satisfactory goodness of fit (R^2^Y = 0.92, Q^2^Y = 0.82, **Figure S3 d**), demonstrating an obvious metabolic perturbation in the PMCAO rats. Loading plots and S-plots also indicated that PMCAO group had serious disorders of metabolites. The color-coded table revealed that PBBG2/PBBG4 only significantly reversed a fraction of metabolites in the brain resulting from PMCAO, which may contribute to its poor effects on reducing brain injury **(Table S4)**.

**Supplementary Figures**


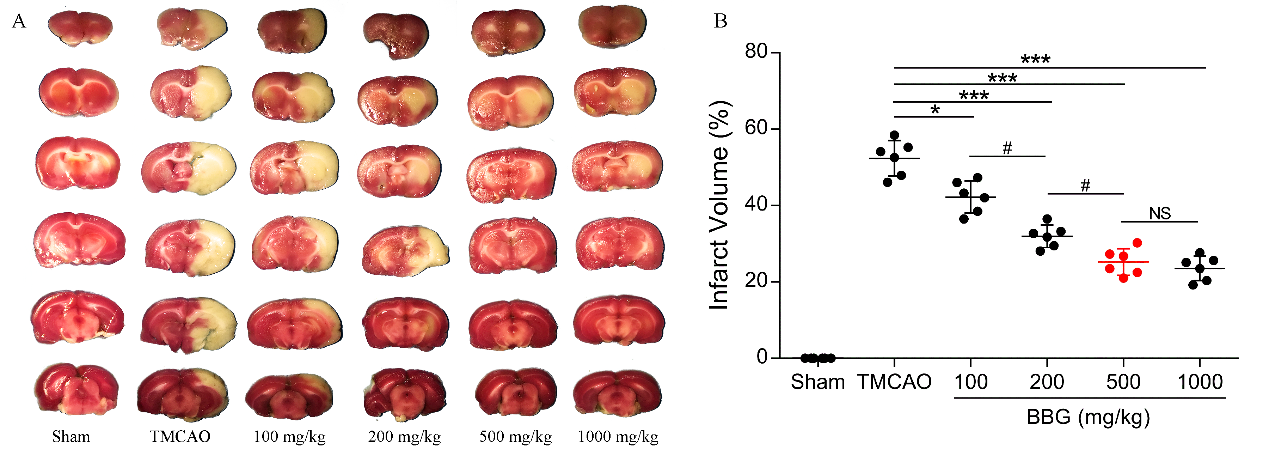


**Figure S1.** Comparison of infarct volumes among different doses of BBG. TTC staining of brains from Sham, TMCAO, 100 mg/kg BBG, 200 mg/kg BBG, 500 mg/kg BBG, and 1000 mg/kg BBG groups (A). The infarct volumes were further calculated (B).


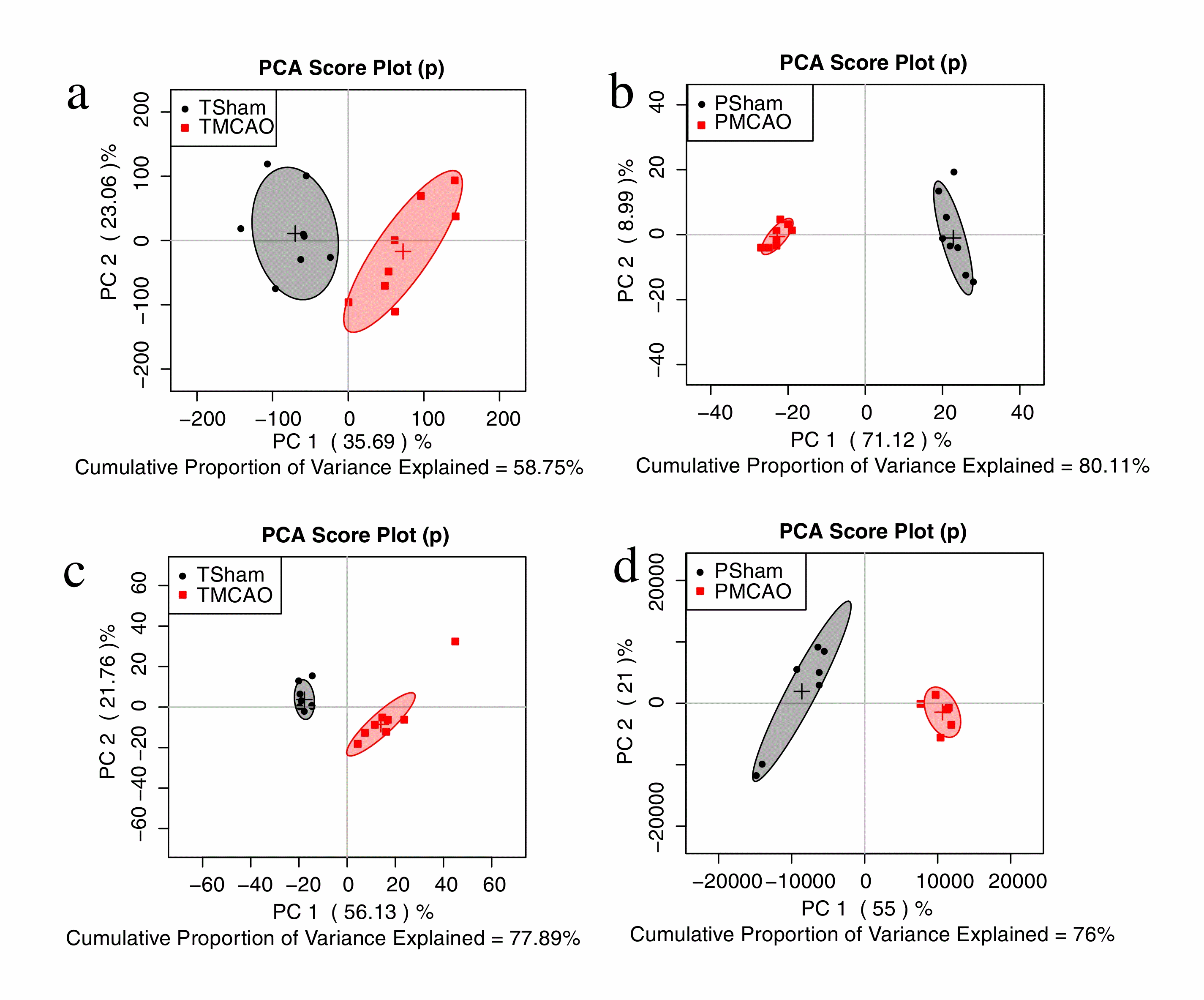


**Figure S2**. The PCA score plots between Sham group and MCAO group. The results between Sham group and TMCAO/PMCAO group were based on NMR (a and b are for serum, c and d are for brain tissue).


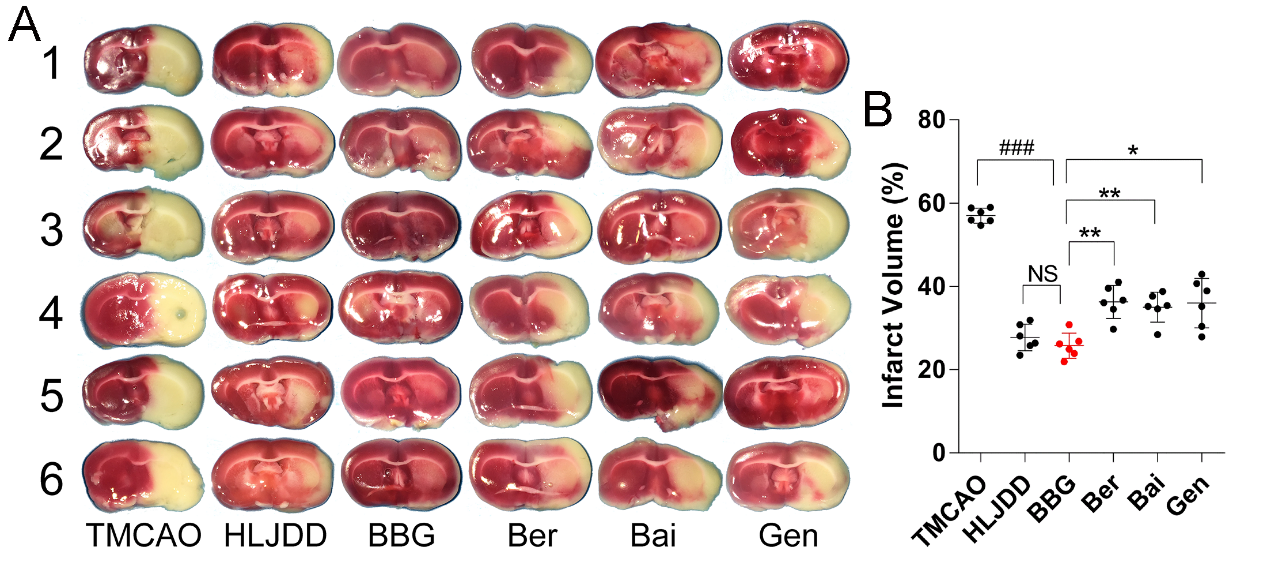


**Figure S3.** Comparison of infarct volumes between BBG and HLJDD treatment groups. TTC staining of brains from TMCAO, HLJDD, BBG, berbine, baicalin and geniposide groups (A). The infarct volumes of each group were calculated (B).

**Figure S4.** OPLS-DA scatter plots of statistical validation obtained by 2,000 times permutation tests between Sham group and MCAO group. The tests between Sham group and TMCAO (a and c) or PMCAO (b and d) group were based on NMR (a and b are for serum, c and d are for brain tissue). With R^2^ and Q^2^ values in the vertical axis, the correlation coefficients (between the permuted and true class) in the horizontal axis, and the ordinary least squares (OLS) line for the regression of R^2^ and Q^2^ on the correlation coefficients.


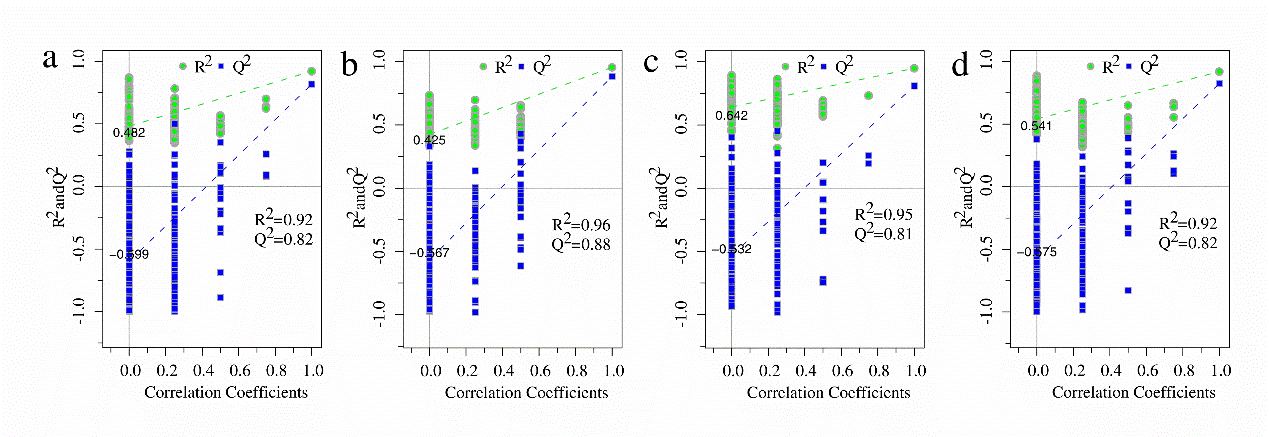

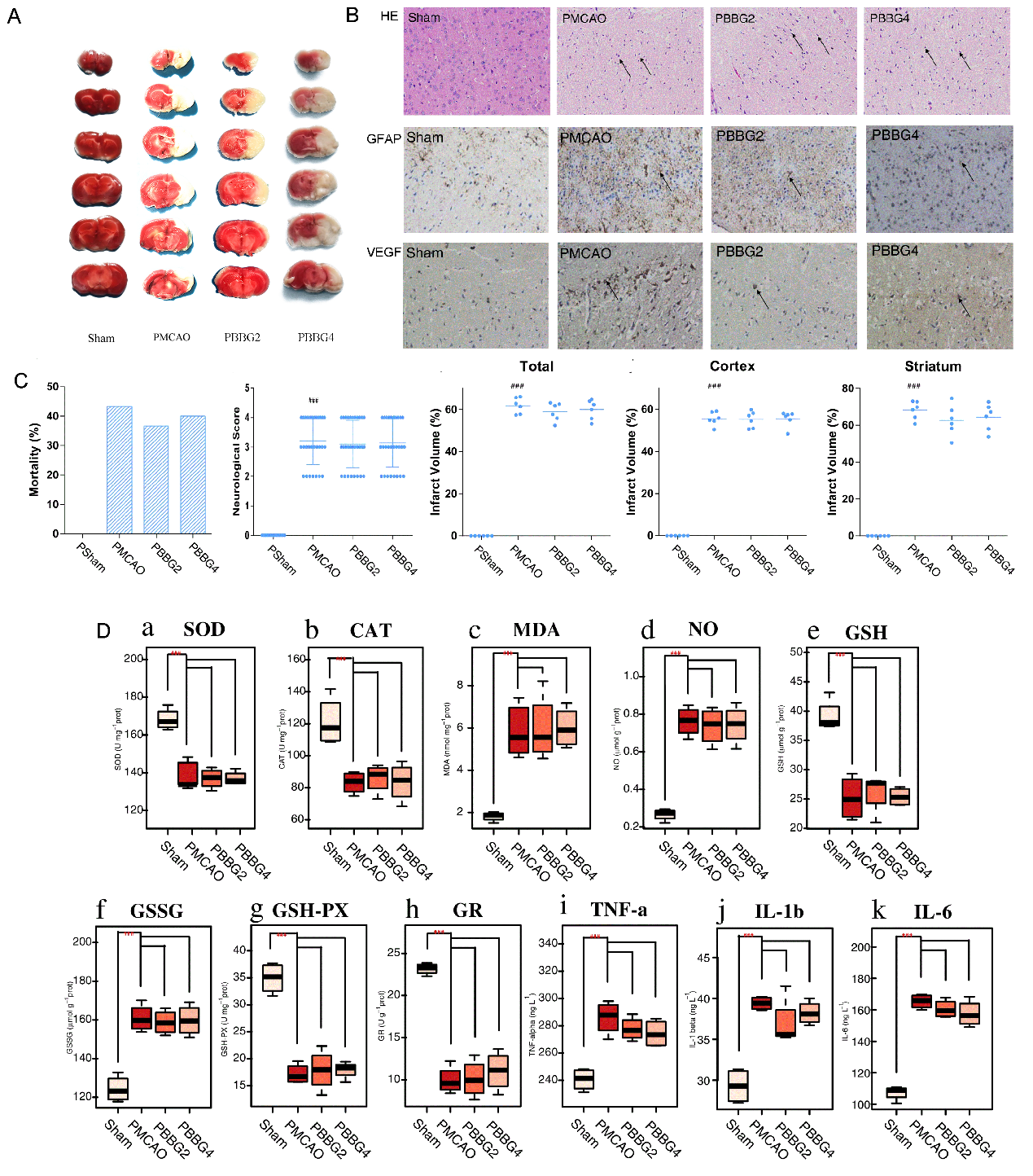


**Figure S****5.** Assessment of neuroprotective effects for BBG in PMCAO rats. TTC staining of brains (n=6) (A). HE staining and immunohistochemical staining of brain tissues to reflect neuronal loss and expressions of GFAP and VEGF (× 200, n=4) (B). Morality, neurological score and infarct volume of each group (C). Boxplots for oxidative stress indexes and inflammatory cytokines (D). #: p < 0.05, ^##^: *p* < 0.01, and ^###^: *p* < 0.001 PMCAO group vs. Sham group; *: *p* < 0.05, **: *p* < 0.01, and ***: *p* < 0.001 treatment groups vs. PMCAO group.


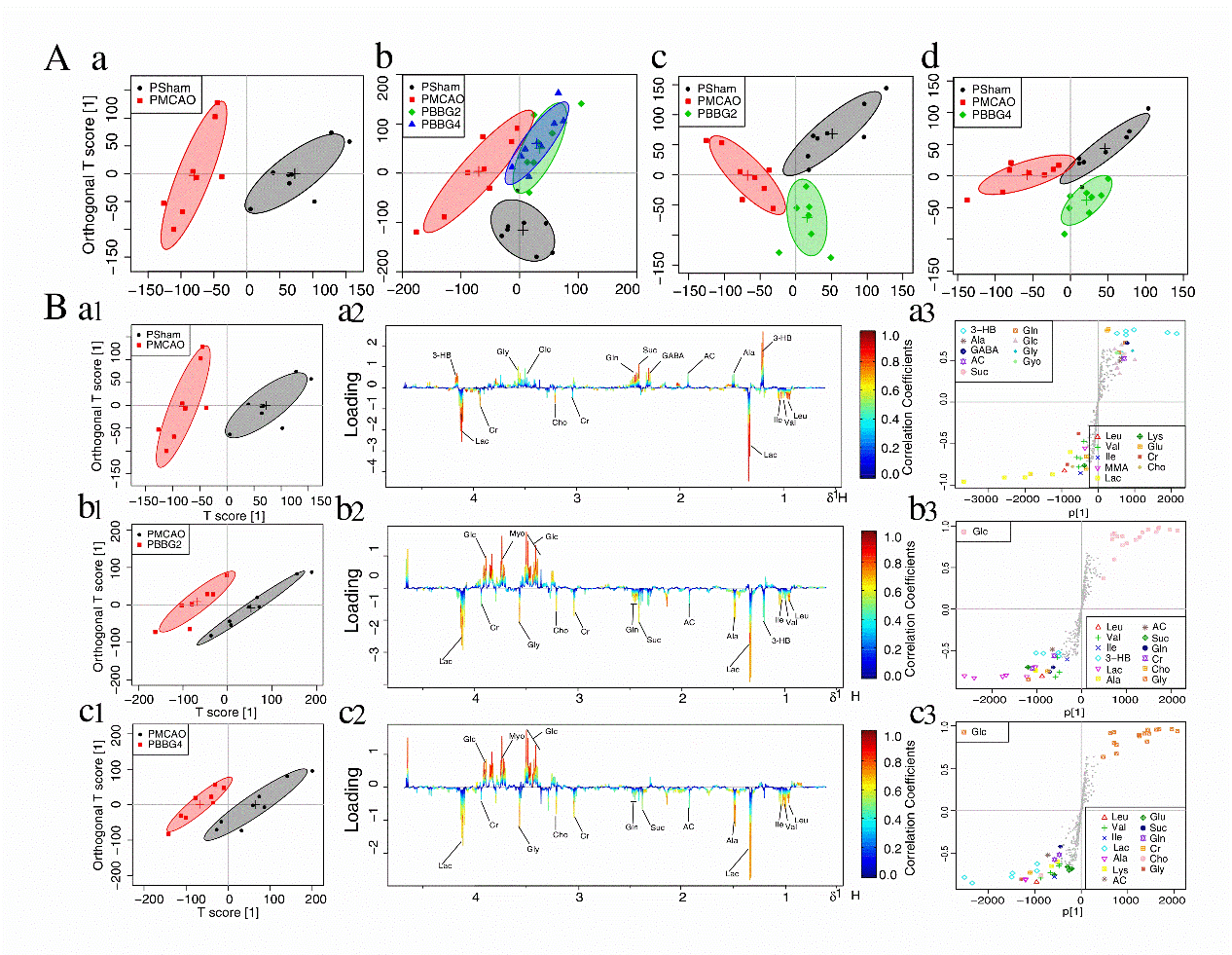


**Figure S6.** OPLS-DA analysis of serum from PMCAO treatment groups based on 1H NMR data. Score plots of OPLS-DA analysis based on 1H NMR from serum samples of Sham (PSham), PMCAO, PBBG2, and PBBG4 groups (n=8) (A). Color-coded loading plots and S-plot for OPLS-DA analysis in serum samples (B).


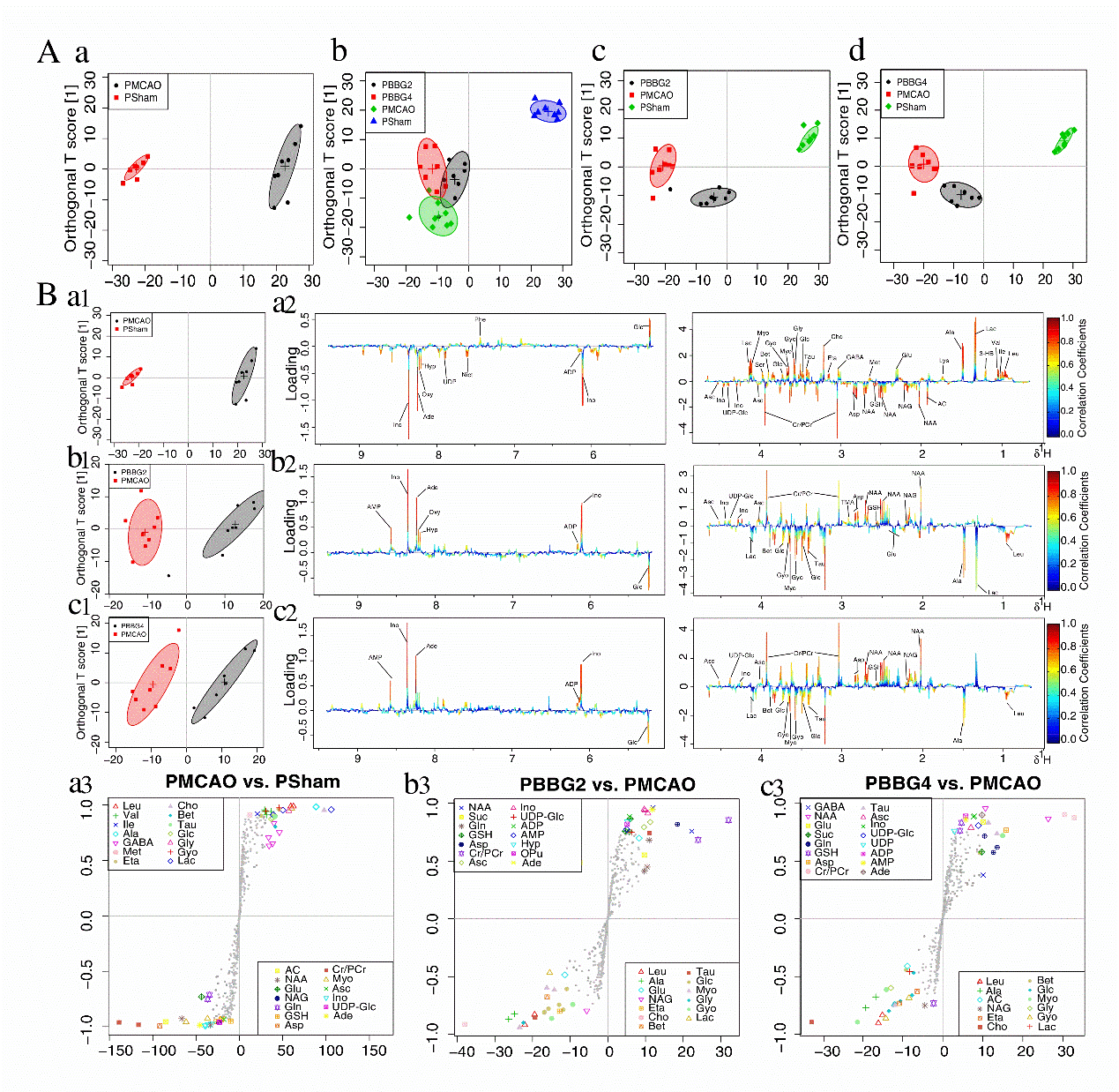


**Figure S7.** OPLS-DA analysis of brains from PMCAO treatment groups based on 1H NMR data. Score plots of OPLS-DA analysis based on 1H NMR from brain samples of Sham (PSham), PMCAO, PBBG2, and PBBG4 groups (n=8) (A). Color-coded loading plots and S-plot for OPLS-DA analysis in serum samples (B).


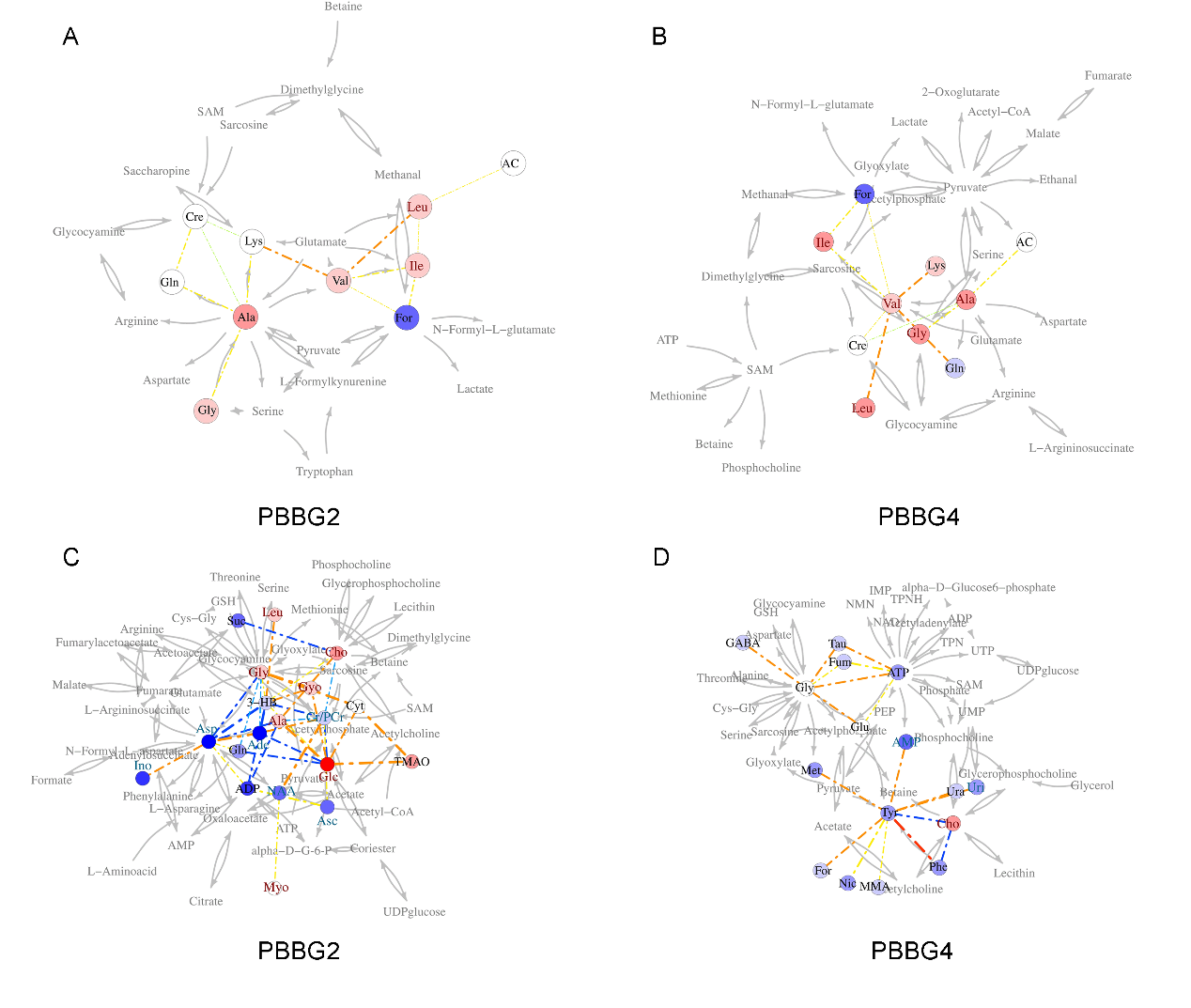


**Figure S8.** Correlation network of BBG treatment in PMCAO rats. Network analysis of PBBG2 and PBBG4 treatment groups in serum (A-B) and ischemic brain (C-D). The networks were constructed by connecting metabolites with dotted lines colored warm or cool to represent positive or negative correlation. The width represented absolute values of coefficients. The warm or cool color of metabolites represented obvious increased or decreased levels in each treatment groups.

**Supplementary Tables**

**Table S1.** ^1^H NMR assignment of metabolites in serum.

| **Metabolites** | **Assignments** | **Chemical shifts** | **Shift range** | **Shift range** |
| --- | --- | --- | --- | --- |
| Leucine | δCH_3_ | 0.96 (d), 0.97 (d) | 0.960-0.982 |  |
| Valine | δCH_3_, γCH_3_, αCH | 0.94 (t), 1.01 (d), 3.66 (d) | 0.984-1.006 | 1.035-1.060 |
| Isoleucine | γCH_3_, γCH_3_, αCH | 1.00 (d), 1.05 (d), 3.62 (d) | 1.006-1.030 |  |
| 3-Hydroxybutyrate | CH_3_, CH_2_ | 1.20 (d), 2.41 (dq) | 1.192-1.216 | 2.390-2.402 |
| Methylmalonate | CH_3_ | 1.22(d) | 1.216-1.235 |  |
| Lactate | CH_3_, CH | 1.33 (d), 4.12 (q) | 1.315-1.35 | 4.09-4.137 |
| Alanine | βCH_3_, αCH | 1.49 (d), 3.78 (q) | 1.47-1.502 |  |
| Lysine | δCH_2_, βCH_2_, εCH_2_ | 1.72 (m), 1.91 (m), 3.30 (t) | 1.70-1.77 |  |
| GABA | βCH_2_, αCH_2_ | 1.91 (m), 2.30 (t), 3.01 (t) | 1.87-1.9074 | 2.286-2.295 |
| Acetate | CH_3_ | 1.92 (s) | 1.9074-1.931 |  |
| Glutamate | βCH_2_, γCH_2_ | 2.11 (m), 2.35 (m) | 2.016-2.065 | 2.332-2.375 |
| Pyruvate | CH_3_ | 2.38(s) | 2.375-2.383 |  |
| Succinate | O=C-CH_2_-CH_2_-C=O | 2.41(s) | 2.402-2.416 |  |
| Glutamine | βCH_2_, γCH_2_, αCH | 2.14 (m), 2.46 (m), 3.78 (t) | 2.442-2.485 |  |
| Citrate | 1/2CH_2_, 1/2CH_2_ | 2.53 (d), 2.65 (d) | 2.515-2.56 | 2.652-2.695 |
| Creatine | CH_3_, CH_2_ | 3.04 (s), 3.93 (s) | 3.035-3.048 | 3.93-3.943 |
| Ethanolamine | CH_2_ | 3.14 (t) | 3.134-3.170 |  |
| Choline | CH_3_ | 3.21 (s) | 3.205-3.216 |  |
| Glucose | CH | 3.3-3.7 (m) | 3.38-3.558 | 3.870-3.925 |
| Glycine | CH_2_ | 3.56 (s) | 3.56-3.57 |  |
| Glycerol | O-CH | 3.58(dd), 3.66(dd) | 3.636-3.674 |  |
|  |  |  |  |  |

**Table S2.** ^1^H NMR assignment of metabolites in brain.

| **Metabolites** | **Assignments** | **Chemical shifts** | **Shift range** | **Shift range** | **Shift range** |
| --- | --- | --- | --- | --- | --- |
| Leucine | δCH_3_ | 0.96 (d), 0.97 (d) | 0.95-0.983 |  |  |
| Valine | δCH_3_, γCH_3_, αCH | 0.94 (t), 1.01 (d), 3.66 (d) | 0.984-1.006 | 1.033-1.065 |  |
| Isoleucine | γCH_3_, γCH_3_, αCH | 1.00 (d), 1.05 (d), 3.62 (d) | 1.006-1.032 |  |  |
| 3-Hydroxybutyrate | CH_3_, CH_2_ | 1.20 (d), 2.41 (dq) | 1.19-1.215 |  |  |
| Methylmalonate | CH_3_ | 1.22(d) | 1.215-1.235 |  |  |
| Alanine | βCH_3_, αCH | 1.49 (d), 3.78 (q) | 1.47-1.502 |  |  |
| Lysine | δCH_2_, βCH_2_, εCH_2_ | 1.72 (m), 1.91 (m), 3.30 (t) | 1.701-1.757 |  |  |
| GABA | βCH_2_, αCH_2_ | 1.91 (m), 2.30 (t), 3.01 (t) | 1.87-1.915 | 2.280-2.320 | 2.99-3.033 |
| Acetate | CH_3_ | 1.92 (s) | 1.915-1.928 |  |  |
| N-Acetylaspartate | CH_3_ | 2.02 (s), 2.68 (dq) | 2.014-2.028 | 2.517-2.525 | 2.713-2.720 |
| Glutamate | βCH_2_, γCH_2_ | 2.11 (m), 2.35 (m) | 2.064-2.100 | 2.330-2.380 |  |
| N-Acetylglutamate | O=C-CH_2_ | 2.21(t) | 2.202-2.226 |  |  |
| Succinate | O=C-CH_2_-CH_2_-C=O | 2.41(s) | 2.404-2.418 |  |  |
| Glutamine | βCH_2_, γCH_2_, αCH | 2.14 (m), 2.46 (m), 3.78 (t) | 2.432-2.472 |  |  |
| Citrate | 1/2CH_2_, 1/2CH_2_ | 2.53 (d), 2.65 (d) | 2.531-2.537 | 2.650-2.656 |  |
| GSH | -N-CO-CH_2_, | 2.56 (ddt), S-CH_2_ (dddd) | 2.54-2.61 | 2.93-2.96 |  |
| Methionine | -S-CH_2_ | 2.64 (t) | 2.625-2.650 |  |  |
| Aspartate | CH_2_, CH | 2.70(dd), 2.82(dd), 3.90(dd) | 2.804-2.814 | 2.834-2.841 |  |
| TMA | CH_3_ | 2.9 (s) | 2.888-2.896 |  |  |
| Creatine/Creatine phosphate | CH_3_, CH_2_ | 3.04 (s), 3.93 (s) | 3.033-3.052 | 3.925-3.95 |  |
| Malonate | CH_2_ | 3.11 (s) | 3.095-3.115 |  |  |
| Ethanolamine | CH_2_ | 3.14 (t) | 3.127-3.16 |  |  |
| Choline | CH_3_ | 3.21 (s) | 3.202-3.215 |  |  |
| TMAO | CH_3_ | 3.25 (s) | 3.245-3.255 |  |  |
| Betaine | N(CH_3_)_3_, CH_2_ | 3.27 (s), 3.89(s) | 3.268-3.277 | 3.886-3.896 |  |
| Taurine | N-CH_2_, S-CH_2_ | 3.27 (t), 3.42 (t) | 3.41-3.45 |  |  |
| Glucose | CH | 3.3-3.7 (m) | 3.45-3.508 | 3.7-3.73 |  |
| Myo-inositol | CH | 3.54 (dd), 3.63 (t), 4.07 (t) | 3.522-3.555 | 3.605-3.646 | 4.058-4.076 |
| Glycine | CH_2_ | 3.56 (s) | 3.560-3.570 |  |  |
| Glycerol | O-CH | 3.58(dd), 3.66(dd) | 3.573-3.583 | 3.656-3.671 |  |
| Threonine | αCH, βCH | 3.59 (d), 4.24 (m) | 3.585-3.593 | 4.255-4.264 |  |
| Serine | O-CH_2_ | 3.96(m) | 3.953-3.971 |  |  |
| Ascorbate | O-CH, ring-CH | 4.02(m), 4.52 (d) | 4.01-4.044 | 4.512-4.530 |  |
| Lactate | CH_3_, CH | 1.33 (d), 4.12 (q) | 4.09-4.14 |  |  |
| O-phosphocholine | N-CH_3_ | 3.22 (s) | 3.22-3.232 | 4.157-4.205 |  |
| Inosine | 5-CH, 4-CH, 2-CH | 4.29(q), 4.45(q), 6.1(d) | 4.272-4.29 | 4.43-4.462 | 6.09-6.125 |
| UDP-glucose | O-CH-O, -CH= | 5.63 (s), 5.95 (dd) | 4.382-4.395 | 5.610-5.643 |  |
| Uracil | CH | 5.81 (d), 7.55 (d) | 5.79-5.825 | 7.529-7.557 |  |
| Uridine | CH-N, N-CH= | 5.90 (d), 5.92 (d), 7.88 (d) | 5.89-5.916 | 7.87-7.905 |  |
| Cytidine | 10-CH, 11-CH | 6.07 (d), 7.85 (d) | 6.05-6.077 | 7.834-7.866 |  |
| ADP | 2-CH, 7-CH | 6.16 (d), 8.59 (s) | 6.125-6.145 | 8.565-8.585 |  |
| AMP | 2-CH, 12-CH, 7-CH | 6.16 (d), 8.26 (s), 8.61 (s) | 6.145-6.167 | 8.594-8.604 |  |
| Fumarate | CH | 6.52 (s) | 6.520-6.528 |  |  |
| Tyrosine | 2-CH, 6-CH, 3-CH, 5-CH | 6.90 (d), 7.19 (d) | 6.890-6.916 | 7.184-7.211 |  |
| Anserine | 5-CH | 7.11 (s) | 7.155-7.175 |  |  |
| Phenylalanine | benzene ring | 7.38 (m) | 7.3-7.46 |  |  |
| Nicotinurate | 5-CH, 6-CH, 2-CH | 7.60 (q), 8.71 (d), 8.94 (d) | 7.579-7.620 | 8.70-8.73 | 8.935-8.951 |
| Xanthine | CH | 7.92 (s) | 7.952-7.965 |  |  |
| Guanosine | O-CH-N, N-CH-N | 5.90 (d), 8.01 (s) | 8.00-8.015 |  |  |
| Carnosine | 5-CH, 2-CH | 7.09 (s), 8.12 (s) | 8.115-8.14 |  |  |
| Hypoxanthine | 2-CH, 7-CH | 8.20 (s) | 8.19-8.206 |  |  |
| Oxypurinol | CH | 8.22 (s) | 8.21-8.227 |  |  |
| Adenosine | CH-OH, N=CH-N | 8.25 (s), 8.34 (s) | 8.235-8.25 |  |  |
| NADH | pyrimidine-CH, imidazole-CH | 8.16 (s), 8.42 (s) | 8.42-8.445 |  |  |
| Formate | CH | 8.46 (s) | 8.455-8.465 |  |  |
| ATP | 7-CH | 8.55 (s) | 8.52-8.545 |  |  |

**Table S3**. Potential marker metabolites and their fold changes^a^ among PMCAO treatment groups and the associated P-values^b^ in serum based on ^1^H NMR.

| NO. | Metabolite | PMCAO/PSham | | PBBG2/PMCAO | | PBBG4/PMCAO | |
| --- | --- | --- | --- | --- | --- | --- | --- |
|  |  | FC | P | FC | P | FC | P |
| 1 | Leu | 1.47 | ** | 1.27 | * | 1.35 | * |
| 2 | Val | 1.43 | ** | 1.23 |  | 1.31 | * |
| 3 | Ile | 1.65 | ** | 1.16 | * | 1.49 | ** |
| 4 | 3-HB | 0.46 | ** | 1.86 | * | 1.11 |  |
| 5 | MMA | 1.3 |  | 0.72 |  | 0.58 | * |
| 6 | Lac | 1.24 | ** | 1.1 |  | 1.1 |  |
| 7 | Ala | 0.77 | * | 1.37 |  | 1.43 | * |
| 8 | Lys | 0.93 |  | 1.07 |  | 1.17 |  |
| 9 | GABA | 0.76 | ** | 1.14 |  | 0.94 |  |
| 10 | AC | 0.81 | * | 1.13 |  | 1.06 |  |
| 11 | Glu | 1.17 |  | 1.04 |  | 1.15 | * |
| 12 | Pyr | 1.16 |  | 1.13 |  | 1.77 |  |
| 13 | Suc | 0.55 | * | 1.69 |  | 1.32 |  |
| 14 | Gln | 0.73 | * | 1.06 |  | 0.98 |  |
| 15 | Cit | 0.91 |  | 1 |  | 1.08 |  |
| 16 | Cr | 1.23 |  | 1.12 |  | 1.14 |  |
| 17 | Eta | 0.83 |  | 1.01 |  | 1.1 |  |
| 18 | Cho | 1.58 | * | 1 |  | 1.31 |  |
| 19 | Glc | 0.92 |  | 0.81 |  | 0.89 |  |
| 20 | Gly | 0.68 | ** | 1.31 |  | 1.4 | * |
| 21 | Gyo | 0.73 |  | 1.21 |  | 0.89 |  |

^a^ Color coded according to the fold change (FC), red represents higher and blue represents lower concentrations of metabolites. Color bar


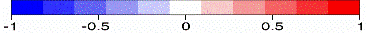


^b^ P-values corrected by Benjamini–Hochberg methods were calculated based on a parametric Student’s t-test or a nonparametric Mann–Whitney test. *p < 0.05, **p < 0.01.

**Table S4.** Potential marker metabolites and their fold changes^a^ among PMCAO treatment groups and the associated P-values^b^ in brain based on ^1^H NMR.

| NO. | Metabolite | PMCAO/PSham | | PBBG2/PMCAO | | PBBG4/PMCAO | |
| --- | --- | --- | --- | --- | --- | --- | --- |
|  |  | FC | P | FC | P | FC | P |
| 1 | Leu | 2.19 | ** | 0.79 | * | 0.87 | ** |
| 2 | Val | 2.06 | ** | 1.04 |  | 0.99 |  |
| 3 | Ile | 1.82 | ** | 1.03 |  | 1.02 |  |
| 4 | 3-HB | 2.14 | ** | 1.04 |  | 0.98 |  |
| 5 | Mma | 1.13 |  | 1.05 |  | 1.03 |  |
| 6 | Ala | 3.11 | ** | 0.78 | * | 0.84 | * |
| 7 | Lys | 1.44 | ** | 1.01 |  | 1 |  |
| 8 | GABA | 1.24 | * | 1.08 |  | 1.11 |  |
| 9 | AC | 0.57 | ** | 0.98 |  | 0.92 |  |
| 10 | NAA | 0.52 | ** | 1.44 | ** | 1.57 | ** |
| 11 | Glu | 0.95 | * | 0.94 |  | 0.96 |  |
| 12 | NAG | 0.27 | ** | 1.17 |  | 1.11 |  |
| 13 | Suc | 0.86 |  | 1.43 |  | 1.5 |  |
| 14 | Gln | 0.69 | * | 1.24 |  | 1.34 |  |
| 15 | Cit | 1.44 | * | 1.03 |  | 1.06 |  |
| 16 | GSH | 0.53 | ** | 1.09 |  | 1.14 |  |
| 17 | Met | 1.94 | ** | 1.2 |  | 1.16 |  |
| 18 | Asp | 0.19 | ** | 2.66 | * | 2.16 | * |
| 19 | TMA | 0.56 | ** | 1.51 | * | 1.36 | * |
| 20 | Cr/PCr | 0.63 | ** | 1.12 | * | 1.12 | * |
| 21 | Mal | 0.58 | ** | 1.14 |  | 1.07 |  |
| 22 | Eth | 1.84 | ** | 0.9 | * | 0.9 | * |
| 23 | Cho | 2.21 | ** | 0.71 | ** | 0.73 | ** |
| 24 | TMAO | 2.2 | * | 0.71 |  | 0.95 |  |
| 25 | Bet | 1.22 | * | 0.83 |  | 0.96 |  |
| 26 | Tau | 1.01 |  | 0.94 |  | 1.02 |  |
| 27 | Glc | 4.75 | ** | 0.41 | * | 0.68 |  |
| 28 | Myo | 0.91 | * | 0.92 | * | 0.96 |  |
| 29 | Gly | 1.39 | ** | 0.85 | * | 0.87 |  |
| 30 | Gyo | 2.59 | ** | 0.77 | * | 0.73 | ** |
| 31 | Thr | 1.13 |  | 1.17 |  | 1.06 |  |
| 32 | Ser | 1.74 | ** | 0.95 |  | 0.92 |  |
| 33 | Asc | 0.63 | ** | 1.36 | * | 1.3 |  |
| 34 | Lac | 2.06 | ** | 0.92 |  | 0.94 |  |
| 35 | OPC | 1.23 | * | 1.02 |  | 0.94 |  |
| 36 | Ino | 0.26 | ** | 1.6 | ** | 1.38 | * |
| 37 | UDP-Glc | 0.43 | ** | 1.35 | * | 1.63 | * |
| 38 | Ura | 0.95 |  | 1.12 |  | 1.11 |  |
| 39 | Uri | 0.45 | ** | 1.1 |  | 1.24 | * |
| 40 | Cyt | 0.84 |  | 0.97 |  | 1.07 |  |
| 41 | ADP | 0.49 | * | 1.53 |  | 1.74 | * |
| 42 | AMP | 0.67 | ** | 1.27 |  | 1.47 | ** |
| 43 | Fum | 0.94 |  | 1.11 |  | 1.02 |  |
| 44 | Tyr | 1.02 |  | 1 |  | 1.22 |  |
| 45 | Ans | 0.85 |  | 0.97 |  | 1.16 |  |
| 46 | Phe | 0.94 |  | 1.13 |  | 1.15 |  |
| 47 | Nic | 0.37 | ** | 1.12 |  | 1.2 |  |
| 48 | Xan | 0.5 | ** | 1.27 |  | 1.27 |  |
| 49 | Gua | 1.95 |  | 1.63 |  | 1.59 |  |
| 50 | Carn | 0.89 |  | 1.04 |  | 1.19 |  |
| 51 | Hyp | 0.35 | ** | 1.53 | ** | 1.28 |  |
| 52 | Oxy | 0.38 | ** | 1.5 | ** | 1.23 |  |
| 53 | Ade | 0.16 | ** | 2.28 | ** | 2.1 | ** |
| 54 | NADH | 0.98 |  | 0.97 |  | 1.07 |  |
| 55 | For | 0.96 |  | 1.01 |  | 1.04 |  |
| 56 | ATP | 0.71 | * | 1.22 |  | 1.26 |  |

^a^ Color coded according to the fold change (FC), red represents higher and blue represents lower concentrations of metabolites. Color bar


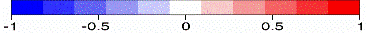


^b^ P-values corrected by Benjamini–Hochberg methods were calculated based on a parametric Student’s t-test or a nonparametric Mann–Whitney test. *p < 0.05, **p < 0.01.
